# Single cell atlas identifies lipid-processing and immunomodulatory endothelial cells in healthy and malignant breast

**DOI:** 10.1038/s41467-022-33052-y

**Published:** 2022-09-20

**Authors:** Vincent Geldhof, Laura P. M. H. de Rooij, Liliana Sokol, Jacob Amersfoort, Maxim De Schepper, Katerina Rohlenova, Griet Hoste, Adriaan Vanderstichele, Anne-Marie Delsupehe, Edoardo Isnaldi, Naima Dai, Federico Taverna, Shawez Khan, Anh-Co K. Truong, Laure-Anne Teuwen, François Richard, Lucas Treps, Ann Smeets, Ines Nevelsteen, Birgit Weynand, Stefan Vinckier, Luc Schoonjans, Joanna Kalucka, Christine Desmedt, Patrick Neven, Massimiliano Mazzone, Giuseppe Floris, Kevin Punie, Mieke Dewerchin, Guy Eelen, Hans Wildiers, Xuri Li, Yonglun Luo, Peter Carmeliet

**Affiliations:** 1 Laboratory of Angiogenesis and Vascular Metabolism, Center for Cancer Biology (CCB), VIB, Department of Oncology, Leuven Cancer Institute (LKI), KU Leuven, Leuven, 3000 Belgium; 2 Laboratory for Translational Breast Cancer Research, Department of Oncology, KU Leuven, Leuven, Belgium; 3 Department of Pathology, University Hospitals Leuven, Leuven, Belgium; 4 Department of General Medical Oncology and Multidisciplinary Breast Centre, University Hospitals Leuven, Leuven, Belgium; 5 Department of Gynaecologic Oncology, Leuven Cancer Institute, University Hospital Leuven, KU Leuven, Leuven, Belgium; 6 Translational Cancer Research Unit, GZA Hospitals Sint-Augustinus, Antwerp, Belgium and Center for Oncological Research, University of Antwerp, Antwerp, Belgium; 7 Multidisciplinary Breast Centre and Department of Surgical Oncology, University Hospitals Leuven, KU Leuven, Leuven, Belgium; 8 Laboratory of Translational Cell & Tissue Research, Department of Imaging and Pathology, KU Leuven, Leuven, 3000 Belgium; 9 State Key Laboratory of Ophthalmology, Zhongshan Ophthalmic Center, Sun Yat-Sen University, 510275 Guangzhou, Guangdong People’s Republic of China; 10 Aarhus University of Advanced Studies (AIAS), Aarhus University, Aarhus, 8000 Denmark; 11 Department of Biomedicine, Aarhus University, Aarhus, 8000 Denmark; 12 Steno Diabetes Center Aarhus, Aarhus University Hospital, Aarhus, 8000 Denmark; 13 Laboratory of Tumor Inflammation and Angiogenesis, Center for Cancer Biology, Department of oncology, KU Leuven, Leuven, 3000 Belgium; 14 Laboratory of Tumor Inflammation and Angiogenesis, Center for Cancer Biology, VIB, Leuven, 3000 Belgium; 15 Lars Bolund Institute of Regenerative Medicine, Qingdao-Europe Advanced Institute for Life Sciences, BGI-Shenzhen, Qingdao, 266555 China; 16 Laboratory of Angiogenesis and Vascular Heterogeneity, Department of Biomedicine, Aarhus University, Aarhus, 8000 Denmark; 17 Center for Biotechnology, Khalifa University of Science and Technology, Abu Dhabi, United Arab Emirates; 18 Present Address: Institute of Biotechnology of the Czech Academy of Sciences, BIOCEV, Vestec, Prague-West Czech Republic; 19 Present Address: National Center for Cancer Immune Therapy, Department of Oncology, University of Copenhagen, Herlev, Denmark

**Keywords:** Breast cancer, Breast cancer, Cancer metabolism, Gene regulation in immune cells

## Abstract

Since a detailed inventory of endothelial cell (EC) heterogeneity in breast cancer (BC) is lacking, here we perform single cell RNA-sequencing of 26,515 cells (including 8433 ECs) from 9 BC patients and compare them to published EC taxonomies from lung tumors. Angiogenic ECs are phenotypically similar, while other EC subtypes are different. Predictive interactome analysis reveals known but also previously unreported receptor-ligand interactions between ECs and immune cells, suggesting an involvement of breast EC subtypes in immune responses. We also identify a capillary EC subtype (LIPEC (Lipid Processing EC)), which expresses genes involved in lipid processing that are regulated by PPAR-γ and is more abundant in peri-tumoral breast tissue. Retrospective analysis of 4648 BC patients reveals that treatment with metformin (an indirect PPAR-γ signaling activator) provides long-lasting clinical benefit and is positively associated with LIPEC abundance. Our findings warrant further exploration of this LIPEC/PPAR-γ link for BC treatment.

## Abstract (web summary)

Tumor blood vessels contribute to cancer growth, invasion and metastasis. Here, by using single cell transcriptomics, the authors report an inventory of endothelial cell heterogeneity in patients with breast cancer, including a subtype that expresses genes involved in lipid processing and is regulated by PPAR-γ.

## Introduction

Breast cancer (BC) affects millions of women worldwide and poses a global health burden. Histological and phenotypical differences between BCs are used to predict outcome and prognosis and guide cancer diagnosis and treatment selection^1^. Importantly, also intra-tumoral heterogeneity in BC might predispose patients to specific clinical outcomes^2,3^. Various single-cell RNA-sequencing (scRNA-seq) studies characterized the inter- and intra-tumoral heterogeneity of BC cells and/or subsets of breast stromal cell types, including immune cells, myoepithelial cells, and fibroblasts^4–10^. Knowledge on vascular heterogeneity in BC, however, has not extended beyond the traditional angiogenic, arterial, capillary, venous, and lymphatic EC phenotypes^11,12^. Nevertheless, obtaining a better understanding of the heterogeneity of the vasculature and its relation to the tumor microenvironment (TME) in BC represents an unmet need, as tumor blood vessels contribute to tumor growth, invasion, and metastasis^13^.

ECs are highly plastic cells^14^ and a target of anti-angiogenic therapies (AATs), based on blocking vascular endothelial growth factor (VEGF) signaling^15,16^. These AATs are used indiscriminately to prune blood vessels in diverse cancer types including BC^17^, but suffer from resistance and insufficient efficacy^16,18^. It remains unknown whether tumor ECs (TECs) from different tumor types exhibit different phenotypes and express distinct transcriptome signatures, which might differentially affect their AAT response. It is also unknown if breast ECs alter their transcriptome in cancer. This might be especially important in the context of EC metabolism (an emerging target of AAT), as recent reports documented that ECs reprogram their metabolism and metabolic transcriptome in pathological conditions such as cancer and ocular disease^14,19,20^. Data comparing the metabolic transcriptome of TECs *versus* peri-tumoral ECs (pECs) in BC are lacking.

In this work, we use single-cell transcriptome data to better understand how ECs might influence BC. Because the large majority of BC patients are diagnosed with early, hormone receptor positive/HER2-negative cancers^21^, we focus on this BC subset and provide a detailed interrogation of EC heterogeneity in matched tumoral and peri-tumoral tissues. Additionally, we explore interactions of ECs with immune cell types. Such cellular interactions are of potential therapeutic relevance e.g., in the light of immunotherapy, since ECs represent a key interface with the immune system^22,23^, but were not studied in detail in previous BC scRNA-seq studies^4–6,24,25^. We also reveal a capillary EC subtype (LIPEC (Lipid Processing EC)), which is enriched in peri-tumoral breast tissues and expresses key regulators of lipid processing (e.g., PPAR-γ). Retrospectively, we reveal a long-lasting positive effect on BC-specific outcome in patients treated with metformin (indirect signaling activator of PPAR-γ), and tumors from metformin-treated BC patients contained more LIPEC vessels than tumors from BC patients who did not receive this treatment.

## Results

### Taxonomy of endothelial cells in the peri-tumoral and tumoral breast

**Fig. 1:**
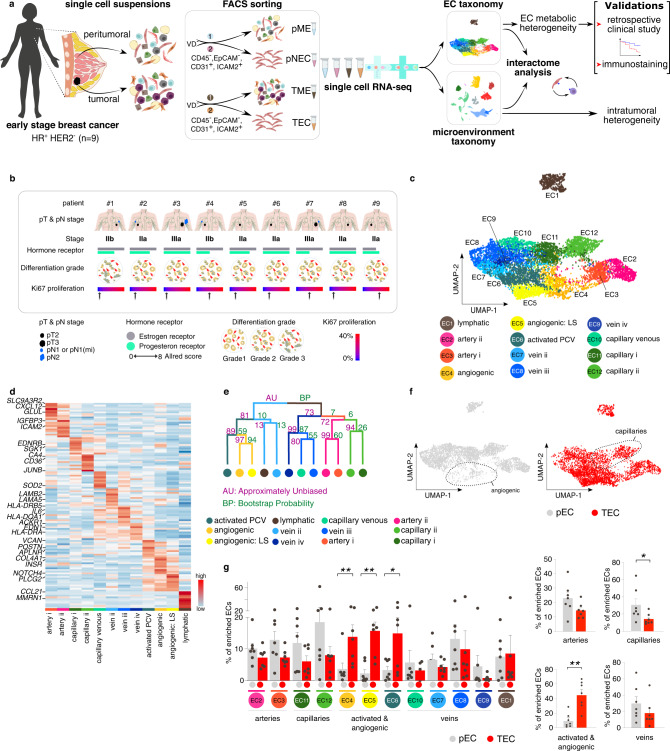
Single cell taxonomy of endothelial cells in the breast. **a** Study design. EC endothelial cell, HR hormone receptor, HER2 human epidermal growth factor receptor 2, VD Viability Dye, pME peri-tumoral microenvironment, pEC peri-tumoral endothelial cells, TME tumor microenvironment, TEC tumor endothelial cells. The numbers related to FACS-sorting indicate sequence of sorting, color coded to indicate that each patient yields 4 samples (pME, pEC, TME, and TEC). **b** Patient characteristics. First line, patient identifier; second line, pathological tumor (pT) and nodal (pN) stage; third line, IUCC (International Union for Cancer Control) cancer stage according to the 8th edition; fourth line, hormone receptor status, coded by color for receptor type (gray, estrogen receptor; green, progesterone receptor) and bar length for Allred score (routinely used immunohistochemistry score based on the percentage of positive cells and the intensity of that staining); fifth line, differentiation grades; grade 1, well differentiated; grade 2, moderately differentiated (patient: #2–8); grade 3, poorly differentiated (patient: #1); sixth line, Ki67 proliferation index based on the clinically performed immunohistochemistry stainings. **c** UMAP-plot, showing the subclustering of 8433 endothelial cells (ECs) from tumoral (*n* = 8) and matched peri-tumoral (*n* = 7) breast cancer (BC) patients. LS lower sequencing depth, PCV post-capillary venules. **d** Heatmap of the expression levels of the top-10 marker genes in all EC subclusters. Color scale: red – high expression, blue – low expression. **e** Hierarchical clustering analysis of EC subclusters. Color differences in the dendrogram indicate clusters that were resolved by multiscale bootstrapping, p-value cutoff 0.4. Approximately unbiased (AU) *p*-values, and bootstrap probability (BP) values are indicated for each dendrogram branch in purple and green, respectively. **f** UMAP-plots of all 8433 ECs color-coded by condition. Dotted lines surround angiogenic (left) and capillary (right) EC subclusters. **g** Abundances of EC subclusters across conditions (pEC (gray), TEC (red)). Left panel: y-axis depicts % of total enriched ECs, *x*-axis depicts EC subtypes color coded as in panel (**c**). Right panels: similar representation of the data as in left panel, but EC subtypes were pooled as indicated. Data are mean ± SEM, *n* = 7 for pEC, *n* = 8 for TEC, *p < 0.05, **p < 0.01 (exact *p*-values = 0.008, 0.0063, 0.0498, respectively (left panel), and 0.0151, 0.0021, respectively (right panel)), paired t-test (two-tailed) per subcluster (taking only into account the 7 complete pairs).

To characterize the transcriptomic heterogeneity of the vasculature and other cell types in human breast (cancer) tissue, we collected tumor tissue and matched (from the same patient) peri-tumoral (non-malignant, as far away from the primary tumor as possible) tissue from 9 early stage, treatment-naïve hormone receptor-positive, HER2-negative BC patients (Fig. 1a) (for patient information and molecular characteristics, see Fig. 1b and Supplementary Data 1). We FACS-sorted viable single cells and divided the freshly isolated cells in two fractions: one used to enrich ECs for a detailed characterization of EC heterogeneity, and the other to study the intercellular interactions between ECs and immune cells (see below). We performed scRNA-seq on TECs and pECs, enriched by FACS-sorting Viability Dye negative (VD^–^)/CD45^–^/EpCAM^–^/CD31^+^/ICAM2^+^ cells from tumoral and peri-tumoral samples (Supplementary Data 2). We compared the EC taxonomy of BC to the in-house generated taxonomy of human lung tumors^19^, which both have a high power and detail of analysis, using the human lung tumor EC taxonomy as a basis to derive the human BC EC taxonomy.

We obtained 8433 high-quality enriched ECs, in which graph-based clustering revealed 12 distinct phenotypes, annotated based on their top-50 marker genes (see “Methods”; Fig. 1c, d; Supplementary Data 3), cluster-specific marker genes (Supplementary Fig. 1a) and previously established gene signatures from in-house generated EC taxonomies^19,26^. Each EC subtype was detected in each sequenced patient sample, though not at the same abundance (Supplementary Fig. 1b).

We identified EC subtypes belonging to traditional vascular beds, i.e. arteries (*HEY1, IGFBP3*), capillaries (*CD36, CA4*), veins (*ACKR1*) and lymphatic ECs (LECs; *CCL21, PROX1*). Arteries, capillaries and veins consisted of multiple subclusters (Fig. 1c, d; Supplementary Fig. 1a; Supplementary Data 3): artery i (*CXCL12*, *AMD1*) & artery ii (*CLU*, *ELN*); capillary i (*ID2*) & capillary ii (*CA4*); and vein ii (*LAMA5*), vein iii (*HLA-DQA1*) & vein iv (*EDN1*)). To obtain independent evidence for the topographical ordering of these traditional EC subtypes alongside the artery-capillary-venous tree, we used in silico lineage tracing to construct in pseudotime a differentiation trajectory (Supplementary Fig. 1c), as previously described^26^. Expression of known arterial (*HEY1, CXCL12*) and venous EC markers (*ACKR1, VCAM1*) was enriched at the opposite ends of the trajectory, whereas capillary markers (*CD36, KDR, EDNRB*) were enriched in the middle of the pseudo-time trajectory (Supplementary Fig. 1d), thus validating our annotation.

A subset of ECs (EC4: angiogenic; EC5: angiogenic LS (LS = lower sequencing depth)) expressed marker genes known to be enriched in tip- and stalk cells *(APLNR, INSR, ESM1*), as well as genes involved in angiogenesis (*KDR, VWA1*) and extracellular matrix (ECM) remodeling (*COL4A1, COL4A2*), as previously described^19^ (Fig. 1d; Supplementary Fig. 1a; Supplementary Data 3)^19^, but proliferating ECs were not detected. Moreover, we identified a subtype of ECs that expressed marker genes of activated post-capillary venules (PCV; EC6; *POSTN*) (Fig. 1d; Supplementary Fig. 1a), previously identified in EC taxonomies of lung cancer and laser-induced choroid neovascularization (CNV)^14,19^ and suggested to represent the vessel type from which neovessels originate^14^. Hierarchical clustering complemented with multiscale bootstrap resampling^27^ confirmed that breast EC subclusters were statistically separable (Fig. 1e). On the dendrogram, angiogenic and activated PCV ECs (EC4–6), involved in vessel sprouting in lung tumors^19^, grouped together, but separately from the traditional arterial, venous, venular (capillary venous) and capillary EC phenotypes (Fig. 1e). Angiogenic and activated PCV ECs (EC4–6) were enriched in TEC samples, while capillaries (EC11–12) were underrepresented (Fig. 1f, g), similar as in lung tumors^19^. Arterial (EC2–3) and venous (EC7–10) ECs were decreased in abundance when comparing TECs to pECs, although not significantly, whereas lymphatic ECs (EC1) were relatively unchanged (Fig. 1f, g). We validated our transcriptome results by immunostaining for selected marker, including INSR (angiogenic ECs), ACKR1 (venous ECs) and CD36 (capillary ECs) (Supplementary Fig. 1e–g). Moreover, we confirmed spatially restricted expression of *ID2* and *FABP4*, top-ranking marker genes of capillary i (EC11) and capillary ii (EC12) subclusters. Conform our scRNA-seq findings, microvascular ECs exclusively expressing *FABP4* or *ID2* (Supplementary Fig. 2a, b) could be identified in human breast (cancer) tissue. Moreover, and in line with the concept of a phenotypic continuum of the EC transcriptome throughout a vascular bed^19^, vessels expressing both *FABP4* and *ID2* could also be detected (Supplementary Fig. 2c).

Next, we used Gene Ontology (GO) enrichment analysis of the top-100 differentially expressed genes (DEGs) of each cluster *versus* all other EC subclusters to obtain insight in their putative biological role (Supplementary Data 3). We observed that different GO terms were enriched in ECs from different vascular beds (Supplementary Fig. 3a; Supplementary Data 3). Arterial ECs (EC3) expressed genes belonging to GO terms involved in cellular junctions and adhesion, whereas angiogenic and activated PCV ECs (EC4–6) were enriched for angiogenic and ECM remodeling processes, in accordance with the literature^19^. Breast capillary ECs were enriched in GO terms involved in lipid metabolism, particularly capillary ii ECs (EC12), which we termed lipid-processing ECs (LIPECs) and will be discussed in a separate paragraph below. Notably, transcripts in breast vein ECs (EC7–10) were enriched in immunoregulatory processes, including regulation of the inflammatory, interferon-gamma, lipopolysaccharide (LPS) and interleukin-1 (IL-1) response (see below for a detailed description). This is in contrast to earlier findings in non-small cell lung cancer (NSCLC) ECs, where EC subtypes with the strongest immunomodulatory signatures were detected in capillaries and PCVs^19^, which prompted us to further explore the differences and similarities in EC subtypes of both cancer types.

### Transcriptome congruency of angiogenic ECs in breast and lung (tumors)

**Fig. 2:**
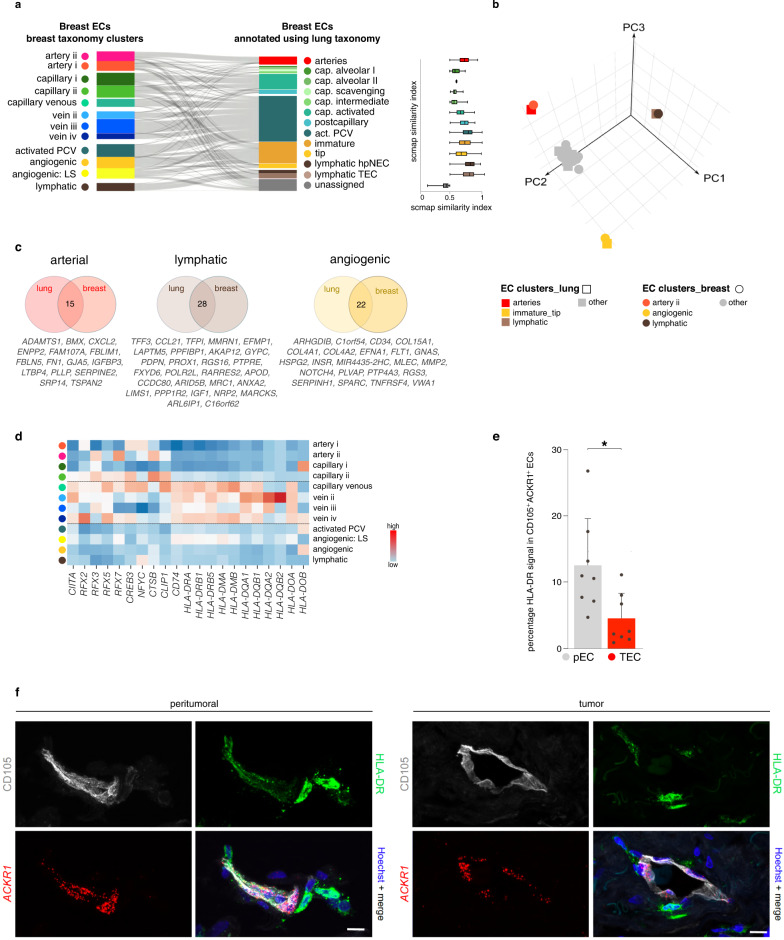
Transcriptomic congruency of ECs in breast and lung. **a** Sankey diagram (left panel), showing the scmap cluster projection of annotated (peri-) tumoral breast-derived ECs (left part; *n* = 8433 endothelial cells) to subclusters of the EC taxonomy in non-small cell lung carcinoma (right part) and box plots (right panel) depicting the scmap similarity index. PCV post-capillary venules, EC endothelial cell, LS lower sequencing depth. Boxes extend from the 25^th^ to 75^th^ percentiles, line in the middle of the box is plotted at the median. Whiskers = min and max. **b** Three-dimensional principal component (PC) analysis on the pairwise Jaccard similarity coefficients of marker genes between EC subtypes in lung and breast. Color coding according to EC subtypes (lung – squares; breast – circles). **c** Venn-diagrams of the top-50 marker genes in the indicated EC subtypes in lung and breast ECs. Numbers in the middle reflect genes congruently ranking in the top-50 in both tissues. **d** Heatmap of the expression levels of the indicated immunoregulatory genes in the different breast EC subtypes. Venous EC subtypes are indicated by dashed lines. Color scale: red – high expression, blue – low expression. **e** Quantification of HLA-DR signal in ACKR1^+^ CD105^+^ pNEC and TEC represented as a percentage of the CD105^+^ vessel area. Data are mean ± SEM, *n* = 7, *p < 0.05 (exact *p*-value = 0.0045), paired t-test (two-tailed). **f** Representative micrographs of human breast peri-tumoral (left) and tumoral (right) tissue sections, immunostained for CD105 and HLA-DR, stained for *ACKR1* by RNAscope and counterstained with Hoechst (*n* = 7). Brightness was decreased linearly (gamma = 1) to improve visibility for *ACKR1* and CD105. Scale bar: 10 µm.

We compared our BC EC taxonomy to a NSCLC EC catalog containing >21,000 ECs^19^ (a publicly available EC dataset with (i) at least equal EC number, quality, and resolution and (ii) both harboring TECs and pECs). Using scmap^28^, we assigned enriched ECs from our BC dataset to the (peri-)tumoral lung EC subclusters^19^. The majority (∼90%) of breast ECs was assigned with high confidence (similarity index >0.5) (Fig. 2a). However, the remaining ∼10% of unassigned breast ECs suggested that our dataset contained breast-specific EC populations (Fig. 2a). Sub-analysis revealed that a large subset (∼40%) of these non-assigned breast ECs were microvascular ECs, of which 76% were LIPECs (Supplementary Fig. 3b). These findings highlight tissue-specific heterogeneity of capillary ECs, consistent with reports that capillaries share few common markers across tissues^26^.

Next, we used the Jaccard similarity index to score the similarity of marker gene sets of all EC subpopulations in breast and lung^19^ (Fig. 2b). This analysis revealed that LECs and angiogenic ECs (referred to as “tip” and “immature” ECs in the lung^19^) in breast and lung tissue resembled each other transcriptomically (Fig. 2a, b). Furthermore, artery ii ECs in the breast resembled arteries in the lung (Fig. 2a, b). In contrast, artery i, capillary, and venous ECs only partially shared an overlapping transcriptome signature between lung and breast (Fig. 2a, b).

Next, we assessed the top-50 ranking marker genes between the angiogenic, lymphatic, and arterial EC clusters in both tumor types in more detail (Fig. 2c). This analysis revealed that in breast- and lung cancer, 22 markers were shared between angiogenic ECs, 28 between LECs and 15 between arterial ECs. Hypothesizing that marker genes, congruently expressed in angiogenic ECs of different cancer types, represent more universal candidate AAT targets than tumor type-specific angiogenic EC markers, we observed that several congruent genes in angiogenic ECs are involved in ECM remodeling (*COL4A1, COL4A2, HSPG2, MMP2, SPARC*; Fig. 2c), as previously suggested in tumor angiogenesis^14,19^. In conclusion, we observed substantial transcriptome overlap of EC phenotypes involved in vessel sprouting in NSCLC and BC, while mainly venous ECs and LIPECs differ, possibly due to EC adaptations to the specific environment in the breast. Therefore, we subsequently focused on those two EC subtypes.

### Vein ECs express immunomodulatory genes

Increasing evidence suggests a possible role for ECs in the perturbed immune homeostasis in cancer^19,29,30^. In agreement, a GO enrichment analysis of our dataset suggested an immunomodulatory role for venous ECs (Supplementary Fig. 3a). Notably, several genes involved in tumor immunity ranked in the top-10 marker genes of vein EC subclusters (Fig. 1d; Supplementary Data 3). Venous ECs (EC7-10) were enriched in genes involved in antigen processing and -presentation, including those important for exogenous antigen presentation (*HLA-II* genes) (Fig. 2d). The co-stimulatory molecules CD80/CD86, critical for naïve T cell activation upon antigen presentation, were undetectable in our dataset, consistent with previous reports in non-breast ECs^19^.

Compared to vein pECs, vein TECs exhibited lower expression levels of a number of genes, involved in antigen presentation (*HLA-DRB1*, *HLA-DRB5,* and *HLA-DRA*)^19,31^, immune cell recruitment (*SELP*, *LIFR*, and *ACKR1*)^32–34^ and anti-tumor inflammation (*CCL14*, *IFITM1*)^35,36^ (Supplementary Fig. 3c). Combined immunostaining for HLA-DR and CD105 (EC marker) with in situ RNAscope hybridization for the venous EC marker *ACKR1*, confirmed lower expression of HLA-DR protein in venous TECs than pECs in *ACKR1*^+^ vessels (for definition of *ACKR1*^+^ vessels see “Methods”; Fig. 2e, f).

### Transcriptome heterogeneity of breast stromal cells

**Fig. 3:**
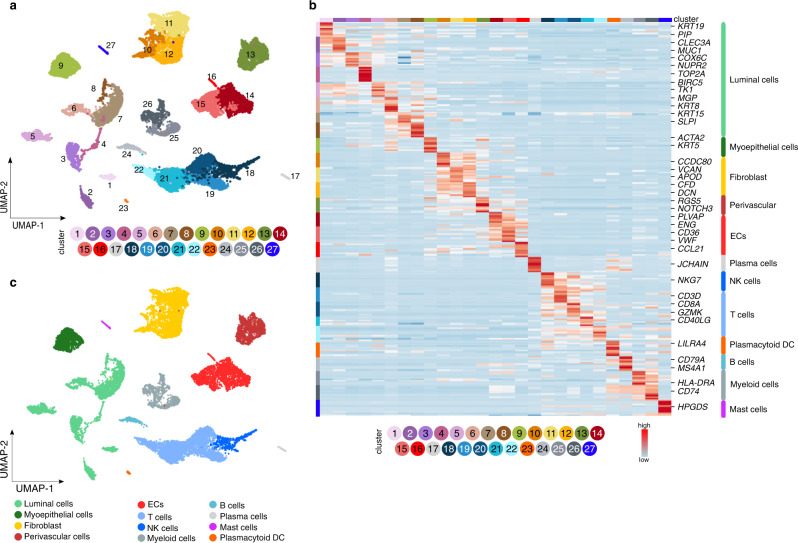
Single cell taxonomy of the breast microenvironment. **a** UMAP-plot of the subclustering of 18,082 cells from tumoral (*n* = 9) and matched peri-tumoral (*n* = 8) BC samples (TME and pTME respectively, composed of all cell types). **b** Heatmap of the expression levels of the top-10 marker genes in all 27 stromal subclusters derived from the non-EC enriched dataset (composed of all cell types). Color scale: red – high expression, blue – low expression. EC endothelial cell, DC dendritic cell, NK natural killer. **c** UMAP-plot of the same cells as in **a**, color-coded for the 12 major cell types.

To obtain more insight in the putative role of ECs in immune responses and how ECs interact with immune cells, we used the breast (cancer) samples without enrichment for any particular cell type to identify microenvironmental (ME) cell types (Fig. 1a). We obtained 18,082 cells and used dimensionality reduction and graph-based clustering to partition the dataset into 27 clusters (ME1–27) (Fig. 3a; Supplementary Data 2). Heatmap analysis of the top-10 ranking marker genes confirmed transcriptome heterogeneity across the different cell clusters (Fig. 3b). Using publicly available taxonomies of cancer- and stromal cell phenotypes in BC^4–6,8,11^, we annotated the 27 ME cell clusters (Fig. 3c; Supplementary Fig. 4a–g; Supplementary Data 4) and identified 12 major cell types within the epithelial (ME1–9), stromal (ME10–16) and immune cell (ME17–27) compartments (Fig. 3b, c), including different types of epithelial cells (luminal and myoepithelial cells), vascular cells (ECs and perivascular cells), fibroblasts and immune cells (plasma cells, NK cells, T cells, plasmacytoid dendritic cells (pDCs), B cells, myeloid cells and mast cells) (Supplementary Fig. 4a–g). Since additional subclustering of epithelial cells (Supplementary Fig. 5a–g, see Supplementary Information for details), perivascular cells (Supplementary Fig. 6a–c) and other stromal cells (mainly fibroblasts; Supplementary Fig. 6d–f) in the (peri-) tumoral breast (Supplementary Data 4) confirmed earlier reports (validating our approach), we did not further focus on these cell types.

**Fig. 4:**
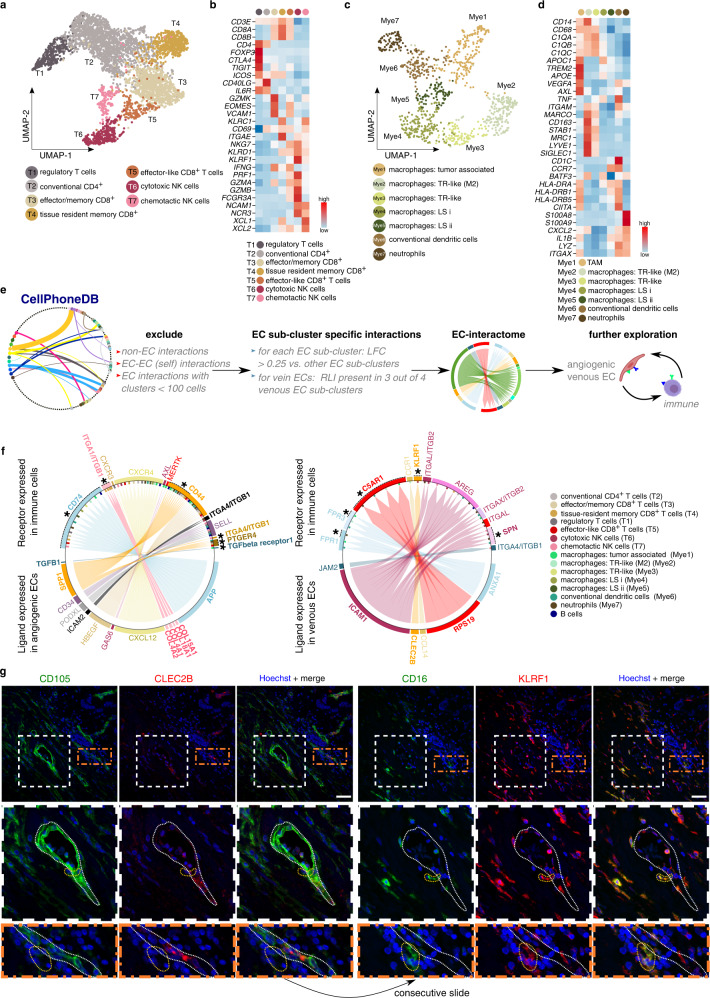
Immune cell subclustering and EC-immune cell interactome predictions. **a** UMAP-plot of T-/NK cells color coded by subcluster. NK natural killer. **b** Heatmap of the expression levels of canonical marker genes of T-/NK cell (sub-)types. Color scale: red – high expression, blue – low expression. **c** UMAP-plot of myeloid cells color coded by subcluster. TR tissue resident, LS lower sequencing depth. **d** Heatmap of the expression levels of canonical genes in myeloid cells. Color scale: red – high expression, blue – low expression. TAM tumor associated macrophages. **e** Schematic overview of the receptor ligand interaction analysis. Clusters containing <100 cells: mast cells, plasma cells and plasmacytoid dendritic cells. EC endothelial cell, LFC log fold change, RLI receptor ligand interaction. **f** Circos plots representing RLI analysis between angiogenic/venous ECs and immune cells. Receptor is expressed on immune cell subclusters, ligand is expressed on angiogenic ECs (left panel) or venous ECs (right panel). Plots are color coded for the receptor–ligand pairs (arrows, gene names) and immune cell subclusters expressing the receptor (bars perpendicular to inner circle). Previously unknown RLI pairs between ECs and specific immune cell subtypes are indicated in bold (genes) and with asterisks (subclusters). **g** Representative micrographs of human breast tumoral tissue sections, immunostained for CD105 and CLEC2B (left panels) or CD16 and KLRF1 (right panels) and counterstained with Hoechst (*n* = 8). Middle panels: magnifications of the white boxed areas in the upper panels. Bottom panels: magnifications of the orange boxed areas in the upper panels. Dotted white line indicates a CLEC2B^+^ blood vessel, dotted orange lines indicate KLRF1^+^ NK cells in the vicinity of the blood vessel. Scale bar: 50 µm.

We used established markers of T cells^9,37^, NK cells^38,39^, and myeloid cells^4,40^ (Fig. 4a–d; Supplementary Fig. 7a–h; Supplementary Data 4, see Supplementary Information for details) to annotate subclusters containing minimally 100 cells. We identified regulatory T cells (T1), conventional CD4^+^ T cells (T2), effector/memory CD8^+^ T cells (T3), tissue resident memory CD8^+^ cells (T4), effector-like CD8^+^ cells (T5), cytotoxic NK cells (T6), and chemotactic NK cells (T7) in the T/NK cell clusters (Fig. 4a, b). We also detected B cells (Fig. 3a–c), which in our dataset were transcriptomically homogeneous. Furthermore, we identified macrophages (Mye1–5), a mixture of conventional dendritic cell subtypes (Mye6; cDC)^41^, and neutrophils (Mye7) (Fig. 4c, d). Macrophage subclusters consisted of tumor-associated macrophages (Mye1; TAMs) and tissue-resident (TR)-like macrophages (Mye2,3). Subclusters Mye4 and Mye5 expressed canonical macrophage genes, but otherwise lacked a distinct transcriptomic phenotype, possibly due to lower sequencing depth (see Supplementary Information). In agreement with literature reports, TAMs (Mye1) expressed a gene signature of both M2-like (anti-inflammatory) and M1-like (pro-inflammatory) macrophages^4,42,43^, while tissue resident macrophage subcluster Mye2 expressed an M2-like signature^4,42,44^, which was less pronounced in tissue resident macrophages from subcluster Mye3 (Fig. 4c, d).

### Endothelial-immune cell interactome predictions

Using CellPhoneDB^45^ and the combined EC-enriched and TME dataset, we characterized the EC interactome by calculating predicted receptor-ligand interaction (RLI) pairs, based on the expression of a receptor by one subcluster and a ligand by another (see “Methods”; Fig. 4e; Supplementary Data 5). We then used various selection criteria to prioritize relevant EC subcluster-specific RLI pairs (see “Methods”; Supplementary Data 5). Since immunomodulatory ECs are receiving increasing attention in scRNA-seq studies, and the potential mechanisms through which ECs can modulate immune cells in healthy and malignant tissues are still being explored^46^, we focused our analysis on the EC-immune cell interactome, and identified multiple immune cell subclusters that were predicted to interact with ECs (Supplementary Data 5). Given their immune-recruiting and -modulatory signature (see above), we focused on venous EC-immune cell interactions. Additionally, given their higher abundance in BC tissue, and the congruency with their counterparts in lung cancer^19^, we also focused on immune cell interactions with angiogenic ECs. We searched the literature to distinguish known from previously unrecognized / poorly characterized interactions and identified—to the best of our knowledge—65 thus far unreported RLI pairs between ECs and immune cell types (Supplementary Data 5; bold, blue), of which we discuss several relevant ones below.

First, we explored the RLI pairs where ECs express the ligand and immune cells the receptor (Fig. 4f). The analysis predicted known interactions that are presumably involved in polarizing macrophages towards an immunosuppressive phenotype by ECs, for instance between angiogenic ECs and TAMs (Mye1) (both enriched in the tumor setting, Supplementary Data 5) expressing respectively *GAS6* (angiogenic ECs) ⇔ *MERTK, AXL* (Mye1, TAMs)^47–49^, suggested to promote immunosuppressive TAM infiltration^50^ (Fig. 4f). Moreover, our RLI analysis predicted tumor-enriched angiogenic ECs and TAMs to interact through *TGFB1* (angiogenic ECs) ⇔ *TGFBR1* (Mye1, TAMs)^51,52^ (Fig. 4f). Interestingly, NicheNet analysis predicted *TGFB1* as the most highly ranked ligand expressed by angiogenic ECs, that likely affects the differential transcriptome in myeloid cells (Supplementary Fig. 7i), indicating that *TGFB1* might indeed be active in modulating myeloid cells, and possibly TAMs, in the tumor. *APP* represents another highly-ranking ligand, and numerous target genes that the NicheNet analysis showed to be regulated by *TGFB1* and *APP* are known to be involved in macrophage polarization towards M1- and M2-like phenotypes (*IL1B*^53^, *IGF1*^54^, *HIF1A*^55^, *CD36*^56^, *TREM2*^57^; Supplementary Fig. 7i). Altogether, this raises the question whether our predicted angiogenic-EC <-> myeloid cells interactions indeed contribute to shaping the tumor immune microenvironment in BC.

Furthermore, the RLI analysis predicted several previously unrecognized / poorly characterized interactions (Fig. 4f, bold, asterisks), thus exemplifying its resource value. As proof of concept, we validated the RLI between PODXL, expressed by angiogenic ECs (marker gene INSR), and its homing receptor L-selectin (SELL) on regulatory T (Treg) cells (marker gene FOXP3; T1) (Fig. 4f; Supplementary Fig. 8a), exemplifying close in situ proximity of the predicted RLI pair. Given its strong enrichment in the tumor setting, and the fact that Treg cells facilitate immune escape and promote angiogenesis^58^, this interaction may warrant further attention. Second, we validated in situ proximity of the ligand CLEC2B (also known as AICL) on venous ECs and the receptor KLRF1 on cytotoxic NK cells (Fig. 4f, g). Venous EC expression of CLEC2B has not yet been documented. However, CLEC2B expressed by myeloid cells has been reported to activate NK cells through interaction with KLRF1, and to enhance NK cell cytolytic capacity and cytokine production^59^, raising the question whether venous ECs might modulate NK cell function. Knockdown (KD) of CLEC2B in human umbilical vein ECs (HUVECs) indeed reduced the percentage of activated human NK cells (freshly isolated from peripheral blood mononuclear cells (PBMCs)) in co-culture experiments (Supplementary Fig. 8b–e), demonstrating a functional role for ECs in NK cell activation.

Subsequently, we focused on interaction pairs where immune cells express the ligand and ECs the receptor (Supplementary Fig. 8f; Supplementary Data 5). This analysis predicted established interactions, for example between *VEGFA* expressed by TAMs (Mye1) and *FLT1/KDR* or *NRP1* on angiogenic ECs, involved in angiogenesis^60^, or between E-selectin (*SELE*) on venous ECs and selectin P ligand (*SELPLG*) on cDCs and TAMs (Mye1, Mye6), involved in immune cell recruitment^61^. The RLI analysis also predicted previously unrecognized / poorly characterized interactions via which immune cells putatively modulate ECs (Supplementary Fig. 8f; bold, asterisks). For example, we identified interactions between angiogenic ECs (*FLT1*, *KDR*, and *NRP1*) and cDCs (*VEGFA*), known to stimulate angiogenesis^62,63^. VEGFA-expressing cDCs are known to promote angiogenesis in inflamed lymph nodes^64,65^, but these interactions with angiogenic ECs were unknown in the tumor context. Importantly, NicheNet analysis predicted cDC-derived *VEGF*-*A* to drive the regulation of genes involved in augmentation of (tumor) angiogenesis, including *S1PR1*^66^, *MMP2*^67^, *PGF*^68^ and *PLPP3*^69^ (Supplementary Fig. 7i). Moreover, NicheNet predicted other cDC-derived ligands (*TGFB1, TNF*) to regulate the angiogenic EC transcriptome (Supplementary Fig. 7i). TNF has a dual effect on angiogenesis^70^, as its promoting/inhitibory effect on angiogenesis is context-dependent^71^. Despite cDCs not being enriched in the tumor setting in our dataset (Supplementary Data 4, 5), these results nevertheless highlight the putative importance of further investigation into cDC-EC crosstalk in light of tumor angiogenesis in BC.

### Heterogeneity of the breast EC metabolic transcriptome

**Fig. 5:**
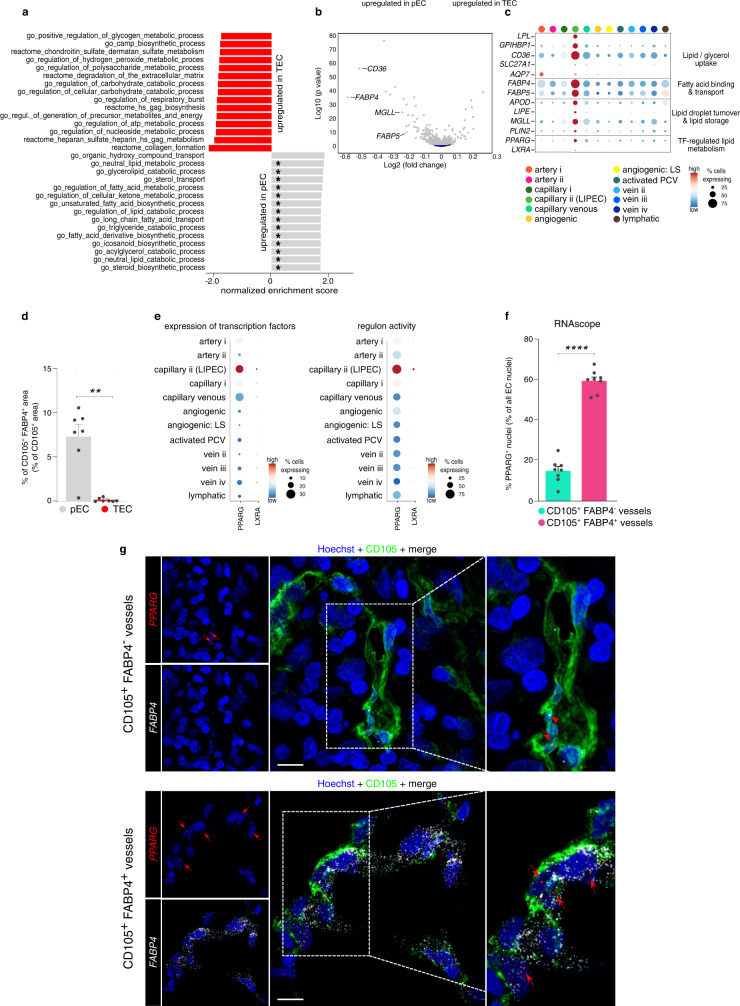
Transcriptomic heterogeneity of breast EC metabolism. **a** Waterfall plot of top-15 up- and downregulated metabolic pathways in metabolic gene set enrichment analysis in TECs compared to pECs (gray – up in pEC, red – up in TEC). Asterisks mark gene sets involved in lipid metabolism. **b** Volcano plot showing differential metabolic gene expression analysis of pECs *versus* TECs. Key pEC-enriched marker genes involved in lipid metabolism are indicated. Gray, significant (adjusted p-value (Benjamini–Hochberg) < 0.05); dark blue, not significant. Differential expression analysis was performed using *limma*, the magnitude of differential expression (log2 fold change) and false discovery rate adjusted *p*-values (Benjamini–Hochberg) are provided on the *x*- and *y*-axis, respectively. **c** Dot plot heatmap of the gene expression levels within the LIPEC signature in breast EC subclusters. The color intensity of each dot represents the average level of marker gene expression, while the dot size reflects the percentage of cells expressing the marker within the subcluster. Color scale: red – high expression, blue – low expression. LIPEC lipid processing EC, TF transcription factor, LS lower sequencing depth. **d** Quantification of the FABP4^+^ CD105^+^ vessel area in peri-tumoral and tumoral breast tissue. Data are mean ± SEM, *n* = 7, **p < 0.01 (exact *p*-value = 0.0014), two-tailed paired t-test. For a representative image of the stained peri-tumoral - tumor border, see Supplementary Fig. 9a. **e** Dot plot heatmap of the expression of *PPARG* and *LXRA* (left panel) and their respective regulons from SCENIC analysis (right panel). The color intensity of each dot represents the average level of gene (left) or regulon (right) expression, while the dot size reflects the percentage of cells expressing the gene/regulon within the cell subcluster. Color scale: red – high expression, blue – low expression. **f** Quantification of the % of EC nuclei with positive *PPARG* staining by RNAscope in CD105^+^ *FABP4*^*+*^ vessels *vs*. CD105^+^ *FABP4*^*−*^ vessels in human breast tissue (tumor and peri-tumoral tissue pooled per patient). Data are mean ± SEM, *n* = 8, ****p < 0.0001 (exact *p*-value < 0.0001), two-tailed paired t-test. **g** Representative micrographs of human breast tumor tissue sections, immunostained for CD105 and stained for *FABP4* and *PPARG* by RNAscope and counterstained with Hoechst (*n* = 8). Right panels: magnifications of the boxed areas in the middle panels. Red arrows point to *PPARG* transcripts stained by RNAscope. Scale bar: 10 µm.

EC metabolism is an emerging target for AAT in several pathological conditions as ECs reprogram their metabolism and metabolic transcriptome during disease^14,19,20^. To explore the metabolic gene signature of breast TECs, we performed a gene set enrichment analysis (GSEA), using an in-house curated list of 910 metabolism-related gene sets selected from the Molecular Signatures Database (complete list in Supplementary Data 3)^72^ in order to compare the metabolic transcriptome landscape of all TECs *versus* all pECs (Fig. 5a). Notably, compared to pECs, TECs were enriched for gene sets involved in ECM remodeling and oxidative phosphorylation, but exhibited lower expression of gene sets involved in lipid metabolism (Fig. 5a, asterisks). Indeed, several of the most DEGs in pECs *versus* TECs are involved in uptake and intracellular transport of fatty acids (*CD36, FABP4, FABP5,* and *MGLL*^73–75^) (Fig. 5b).

We then performed gene set variation analysis (GSVA) on all 12 EC subtypes (both pECs and TECs) (Supplementary Data 3). In angiogenic ECs (EC4–5) and activated PCV ECs (EC6), we predominantly identified gene sets involved in oxidative phosphorylation and ECM remodeling, validating other reports^76^, while venous EC clusters (EC7-9) upregulated gene sets involved in prostaglandin metabolism, in line with their immunomodulatory signature and possible role in vasoregulation, confirming findings in lung tumors and choroid neovascularization^13,19^. Notably, arterial EC clusters (EC2–3) upregulated gene sets involved in the production of the vasoregulator nitric oxide (NO).

In line with the GO enrichment analysis described above, the GSVA revealed an enrichment of gene sets involved in lipid metabolism, transport, and catabolism in breast capillary ii ECs (LIPECs; mainly derived from pECs) (EC12; Supplementary Data 3). Specifically, we observed an enrichment of key regulatory genes involved in lipid and/or glycerol uptake from low-density lipoprotein particles^77^, intracellular binding of free fatty acids^75^, lipid droplet turnover^74^ and transcription factor-mediated regulation of fatty acid metabolism, lipid storage, and sterol homeostasis^78,79^ (Fig. 5c)—based on this signature, we, therefore, termed ECs expressing this signature as LIPECs (see above).

Immunostaining for FABP4 (the most discriminative metabolic gene between TECs and pECs, and enriched in LIPECs (Fig. 5b, c)) revealed that FABP4-expressing capillaries were underrepresented in TECs (Fig. 5d; Supplementary Fig. 9a). Moreover, using Single-Cell Regulatory Network Inference and Clustering (SCENIC)^80^, we detected enriched activity of *PPARG* (also known as *NR1C3*)- and *LXRA* (also known as *NR1H3*)-driven regulons and transcription factor expression in LIPECs (Fig. 5e and Supplementary Fig. 9b). Combined immunostaining for CD105 with in situ hybridization (RNAscope) for *FABP4* and *PPARG* furthermore revealed a significantly higher fraction of *PPARG*^*+*^ ECs within *FABP4*^*+*^ blood vessels, as compared to their *FABP4*^*−*^ counterparts (Fig. 5f, g).

### Lipid processing endothelial cells—possible translational implications?

Interestingly, elevated PPARG (PPAR-γ) expression in BC stromal cells has been reported to be a good positive prognostic marker, associated with longer survival after upfront surgery^81^, but data reporting a possible effect of PPAR-γ agonist treatment on long-term clinical outcome and LIPEC vessel growth in hormone receptor-positive (ER^+^, PR^+/−^)/HER2-negative BC patients are limited. In an exploratory initial study, we assessed whether possible activation of PPAR-γ signaling, indirectly through administration of the biguanide metformin^82,83^ (one of the agents used as standard of care treatment of diabetes mellitus^84,85^): (i) might be involved in clinical outcome of BC patients, and (ii) might be related to LIPEC abundance, realizing that metformin may have additional mechanisms of action.

**Fig. 6:**
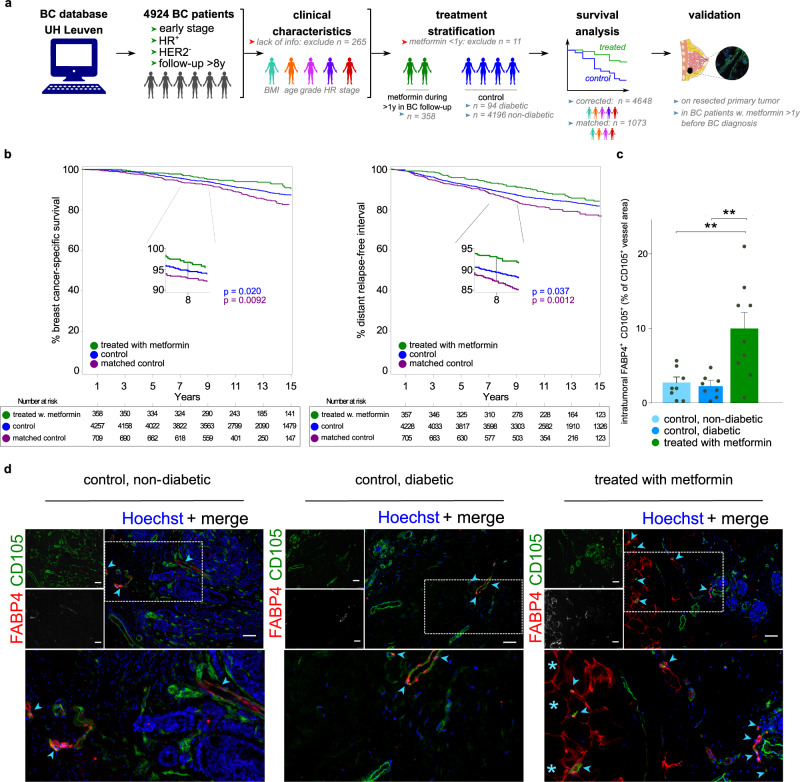
Lipid processing endothelial cells—translational implications. **a** Schematic overview of the survival analysis in the retrospective clinical cohort and immunostaining validation. UH university hospital, BC breast cancer, HER2 human epidermal growth factor receptor 2, BMI body mass index, HR hormone receptor status. Color coding in the clinical characteristics panel reflects differences in age, BMI, tumor stage & grade (Supplementary Data 6). Color coding underneath the treatment stratification panel indicates patients that did (green) or did not (blue) receive metformin treatment during follow up. **b** Cumulative incidence function estimate of BC-specific survival (left panel) and the distant relapse free interval (right panel) in BC patients stratified by intake of a metformin. Color coded by group: blue – control, green – patients treated with metformin, purple – control matched for age, BMI, tumor stage & grade and hormone receptor status. *P*-values were calculated by the Kaplan–Meier (log rank) test between metformin therapy and without metformin therapy groups (blue for unmatched control patients; purple for matched control patients). Numbers in the boxes underneath the curves depict the number of patients per group that are at risk for the event (mortality in left panel, mortality/development of metastasis in right panel) at the indicated time points. **c** Quantification of FABP4^+^ blood vessels (% area of total CD105^+^ blood vessels) in non-diabetic BC patients (*n* = 8) and in diabetic patients without (*n* = 8) or with (*n* = 9) metformin treatment. Data are mean ± SEM, **p < 0.01 (exact *p*-values = 0.0030 and 0.0023, respectively), one-way ANOVA followed by Dunnett’s multiple comparisons test. **d** Representative micrographs of human breast tumor tissue sections in control non-diabetic (left; *n* = 8) or diabetic (middle; *n* = 8) control BC patients and in (diabetic) BC patients treated with metformin (right; *n* = 9), immunostained for CD105, FABP4 and counterstained with Hoechst. Arrowheads denote CD105^+^FABP4^+^ vessels, asterisks denote (putative) adipocytes, which (besides LIPECs) are also positive for FABP4. Brightness was increased linearly (gamma = 1) to improve visibility for CD105 and FABP4. Scale bar: 75 µm.

We therefore took advantage of a large BC patient dataset with long follow-up (8 years or longer) to retrospectively analyze the effect of metformin treatment on the clinical outcome in a cohort of 4648 female patients with early, hormone receptor-positive/HER2-negative BC, who underwent surgery followed by anti-hormonal treatment (thus, tumors with the same setting and molecular characteristics as used in our scRNA-seq cohort) (Fig. 6a; Supplementary Data 1). In our cohort, all treated BC patients were diabetic and treated with the biguanide metformin. Control patients included those that are diabetic but not treated with metformin, but also non-diabetic controls without treatment to increase statistical power (Fig. 6a). The follow-up was at least 8 years (with 50% of BC patients having a follow-up ≥14 years), making this cohort unique in terms of its magnitude and duration of follow-up, as compared to previous cohorts^86,87^.

We examined the relationship between treatment with metformin (at least one year during follow-up after BC surgery) and BC-specific survival (BCSS) or distant relapse free interval (DRFI) using a Cox proportional hazard analysis, after adjusting for clinical characteristics known to affect BCSS/DRFI (Fig. 6a; Supplementary Data 6; “Methods”). We observed a 31.4% reduction in BC-specific mortality and a 25.5% reduction in the development of metastases in BC patients treated with metformin, compared to control patients (hazard ratio (HR) for BCSS: 0.686 (CI: 0.498–0.944); HR for DRFI: 0.744 (CI: 0.564–0.98)) (Fig. 6b, green and blue groups, respectively; Supplementary Data 6). Notably, adjuvant anti-hormonal treatment (the current standard of care in early-stage hormone responsive BC) induces a similar magnitude of effects on BCSS and DRFI^88^.

In a complementary approach to control for potential confounder effects, we compared the BCSS and DRFI in the group of metformin-treated BC patients to a group of non-diabetic, non-metformin treated BC patients, matched for age, body mass index (BMI), histological grade and pathological (tumor and nodal) stage (see “Methods”; Fig. 6b; green and purple groups). Notably, this analysis showed a similar, if not more pronounced, effect in favor of metformin-treated BC patients (Fig. 6b, green and purple groups; Supplementary Data 6), validating our primary unmatched cohort analysis. Overall, metformin treatment in BC patients induced long-lasting clinical benefits. Secondly, to explore whether metformin treatment affected LIPEC vessels, we quantified the percentage of FABP4^+^ LIPEC vessels in 25 BC patients with similar clinical and pathological characteristics (Supplementary Data 1) and derived from the same cohort as used for the survival analysis. Notably, tumors from BC patients, who were treated with metformin prior to tumor removal, contained 3 to 4-fold more FABP4^+^ LIPEC vessels than tumors from BC patients (diabetic or not), who did not receive this treatment (Fig. 6c, d; Supplementary Fig. 9c).

## Discussion

The aim of this study was not to provide yet another descriptive BC taxonomy, but to deliver an in-depth characterization of the EC heterogeneity in the most common human BC type, and to explore the EC-immune cell interactome. This analysis yielded the following insights.

Currently, most human cancers are treated indiscriminately with the same anti-angiogenic strategy, based on targeting VEGF signaling. Yet, ECs exhibit substantial phenotypic heterogeneity between tissues and even across the vascular tree within a single tissue^19,26^. Here, we show that breast and lung tumors seem to rely on the same EC phenotypes to form new blood vessels, but exhibit detectable phenotypic differences in other EC subtypes, especially at the capillary and venous level. This is consistent with our findings in healthy murine tissues that capillaries are transcriptomically more plastic, likely reflecting their phenotypic adaptation to the physiological needs of the tissue they reside in ref. 26.

Emerging evidence indicates that ECs can contribute to the immune response as non-hematopoietic derived cells^89^. We reported that capillaries in human and murine lungs expressed genes involved in antigen processing and presentation, while PCV ECs exhibited gene expression signatures involved in immune cell recruitment^19^. In this study, we documented that vein ECs in the human breast express immunomodulatory genes, illustrating tissue-specific differences. The lung is exposed continuously to airborne pathogens, and this exposure may be maximal at the capillary level, where exchange of gas and other airborne particles/pathogens is highest^90^. In contrast, in the breast, the low blood flow rate and shear stress in veins may possibly favor the interaction with immune cells. Indeed, vein ECs (distinct from high endothelial venules) in murine lymph nodes have immune recruiting properties^91^, suggesting that the immunomodulatory characteristics of vein ECs might warrant further investigation in BC.

Different kinds of immune cells such as neutrophils, dendritic cells, macrophages and lymphocytes (B cells, T cells) are known to stimulate vessel sprouting through production of pro-angiogenic molecules^92,93^. Additionally, perivascular macrophages are known to assist in the fusion of adjacent vessel sprouts to form a perfused vessel^92^. As an in-depth documentation of EC-immune cell interactomes has been lacking to date, our RLI dataset allows for exploration of the plethora of possible molecular signals of these intercellular communications. Our receptor-ligand interactome predictions identified known (validating our approach) but also previously unrecognized/poorly characterized interactions between ECs and immune cells, some of which suggested that ECs might play immunomodulatory roles. For instance, we predicted specific interactions between tumor-enriched angiogenic ECs and Treg cells or cDCs, with potential implications for tumor angiogenesis, and between venous ECs and cytotoxic NK cells, suggesting that venous ECs may play a role in NK cell modulation in BC. Altogether, these predictions (i) raise the question whether the EC/immune cell interactome may regulate tumor immunity and vessel sprouting in BC, possibly via previously unknown interactions with (among others) Treg cells, cDCs or NK cells; and (ii) provide a rich resource to encourage future functional studies on characterizing immunomodulatory functions of ECs as well as reciprocal communication between ECs and immune cells.

We discovered a breast capillary EC population (LIPEC) that expressed markers involved in lipid metabolism and the transcription factor PPAR-γ, encoding a master regulator of lipid metabolism in ECs and other cell types, which upregulates FABP4 expression. By immunostaining, we confirmed that FABP4^+^ ECs co-expressed PPAR-γ. While SCENIC analysis predicted activity of the PPAR-γ regulon in LIPECs, other transcription factors belonging to the PPAR family (*PPARA, PPARB*) or PPAR-γ co-factors (*RXRA, RXRB,* and *PPARGC1B*) and their downstream signals were only minimally detected or absent from our dataset, and not specifically detected in LIPECs, precluding a more elaborate conclusion on the potential functional role of this EC subtype in lipid metabolism. Notably, LIPEC capillaries were underrepresented in BC. Differential gene expression analysis, comparing LIPECs in tumor *versus* peri-tumoral tissues, revealed *HES1* as the only significantly upregulated gene in tumor LIPECs (Supplementary Data 6). HES1 is involved in vascular remodeling and specification of arterial fate in ECs^94^, but also reported as a repressor of PPAR-γ^95,96^, and the role of *HES1* in LIPEC differentiation, molecular makeup (e.g., expression of *FABP4* and other genes involved in lipid metabolism) and function in cancer thus warrants further attention. When analyzing ECs detected in a publicly available scRNA-seq dataset of ovarian, breast and colorectal tumors^97^, LIPECs were mainly detected in breast and ovarian (cancer) ECs, but were not abundantly found in colorectal cancer ECs (Supplementary Fig. 9d). What the functional significance of LIPECs is for BC development or progression, and whether there are microenvironment-specific factors that underlie the presence/absence of LIPECs in tumors from certain tissues, remain outstanding questions requiring further study.

Our study suggests possible translational implications. First, the fact that angiogenic ECs across human (breast and lung) tumor types share a common transcriptome could have implications for the design of future anti-angiogenic therapies, based on targeting congruent markers. Current AAT strategies, based on blocking VEGF signaling, target angiogenic tip and proliferating stalk ECs^17^. However, and in line with other single-cell studies focusing on human cancer^19,97^, we could not detect proliferating ECs in our human breast tumor scRNA-seq dataset. Angiogenic ECs (expressing tip EC markers) were present at only 14%, and it remains to be determined whether these low fractions of angiogenic TECs may explain the relative inefficiency and resistance to VEGF-blockade based AAT. Second, the observation that breast vein ECs might contribute to the immune response in breast tumors deserves further attention. Third, retrospectively, metformin treatment (which may indirectly lead to PPAR-γ signaling activation^82,83^) improved the long-term clinical outcome of BC patients. Fourth, we coincidently observed an abundance of LIPECs in the population treated with metformin. While at this stage, we cannot provide a direct effect of PPAR-γ agonist treatment on the abundance of LIPECs in BC patients, nor a causal role of LIPECs in BC clinical outcome, our findings nevertheless warrant further investigation into the LIPEC phenotype and its role in BC progression and survival. Additionally, our results raise the question whether an increase in LIPEC vessel counts in tumor biopsies in response to metformin treatment could be considered in future prospective randomized trials as a predictive biomarker for long-term clinical benefit. Related hereto, and even more speculative at this stage, an outstanding question is whether strategies stimulating LIPEC vessel growth and/or differentiation might be beneficial for BC patients. Fifth, the reduction in BCSS and DRFI observed after the current standard of care adjuvant treatment with anti-hormonal treatment^81,88^ is of similar magnitude as the reduction in these clinical outcome parameters, observed after treatment of BC patients with metformin.

We acknowledge limitations of our study. First, the biological role of the identified EC subclusters, inferred from the marker gene analysis, is only putative and requires future (experimental) validation. Second, due to tissue digestion optimized for ECs, abundances of other cell types might be altered. Third, as scRNA-seq does not allow sequencing of mature adipocytes^98^, this cell type is lacking from our transcriptomics analyses. Fourth, the contribution of LIPECs and the precise mechanisms of how PPAR-γ regulates lipid metabolism in LIPECs requires further study. Fifth, profiling metabolic pathways at the transcriptional level does not fully represent the intricacy of metabolic fluxes, levels of metabolites and activity of metabolic enzymes at the protein level. However, previous work has shown that gene expression signatures can be (at least in part) predictive of alterations in metabolic fluxes in ECs^20,99^. Nonetheless, these metabolic gene signatures require further functional validation. Sixth, the identified RLI pairs are a prediction and, although we validated two examples at the protein level, these predictions require further validation. Seventh, to confirm the associative correlation in the retrospective study, prospective trials are needed. Finally, we acknowledge that metformin, besides activating PPAR-γ signaling, likely exerts additional activities on other cellular pathways and also on non-EC cell types^100^, which could contribute to the clinical benefit.

Notwithstanding these limitations, our study provides a rich transcriptome resource of EC heterogeneity in peri-tumoral and malignant breast, and a predicted interactome of stromal cells in BC that provides a basis for future research to study their functional role. It also opens new avenues to consider LIPECs for future functional and biomarker analysis.

## Methods

### Ethical approval

#### Tissue samples

The study was approved by the Medical Ethics Committee UZ Leuven (University Hospital Leuven) under protocol number S57123. All tissue samples were obtained under written informed consent and were fully anonymized. Consent to publish relevant clinical information potentially identifying individuals (e.g., age, BMI, histological grade, etc.) was obtained. Participants were not compensated due to the fact that we only used left-over tissue (secondary use of residuary material), which did not require additional patient follow-up or contact.

#### Clinical cohort

All patient data used for retrospective clinical cohort analyses (and validation) were retrieved from the multidisciplinary BC clinic database in the University Hospital (UZ) Leuven, which was approved by the Medical Ethics Committee UZ/KU Leuven under protocol number S63779.

#### HUVECs

Human umbilical vein endothelial cells (HUVECs) were freshly isolated from umbilical cords obtained from multiple donors of unknown sex soon after birth with approval from the Ethics Committee Research UZ/KU Leuven under the approval number S57123, and informed written consent was obtained from the parents of all subjects. HUVECs were used as single-donor cultures, and a minimum of 3 donors was used for all experiments.

### Human tissue processing

Following surgical resection, samples from the invasive tumor front and adjacent healthy breast tissue (as far away from the tumor border as possible) were taken. Samples were minced into <1 mm^3^ pieces and enzymatically digested in a 7 mL digestion medium (KnockOut DMEM, supplemented with penicillin/streptomycin (1×), Antibiotic-Antimycotic (2×), sodium pyruvate (1 mM), MEM NEAAs (1×) (Thermo Fisher Scientific), heparin (10 U/mL)/ECGF (PromoCell), 0.3% collagenase I, 0.1% collagenase II (Thermo Fisher Scientific), DNase (7.5 µM) (Sigma-Aldrich) and dispase (0.25U/mL) (Thermo Fisher Scientific)). Samples were incubated at 37 °C for approximately 60 min. To improve digestion, samples were pipetted up and down every 10 min. Next, 7.5 mL of cold PBS containing 0.5% bovine serum albumin (BSA, Sigma-Aldrich) and 2 µM EDTA were added and the samples were filtered using a 100 μm strainer (Corning). Following centrifugation (300 g) at room temperature (RT) for 4 min, the supernatant was discarded and cells were resuspended in cold PBS containing 0.5% BSA (Sigma-Aldrich) and 2 µM EDTA for further downstream applications.

### Flow cytometry and cell sorting

Single-cell suspensions were stained with viability dye (VD, eFluor™ 506, dilution 1:1000) and fluorescent antibodies (CD45 (PE-Cy7, dilution 1:700), EpCAM (PE, dilution 1:500), CD31 (AF488, dilution 1:100) and CD102/ICAM2 (APC, dilution 1:50)) for 30 min. Then, we FACS-sorted viable single cells (VD^–^) into low-bind Eppendorf tubes containing 200 µL pure FBS and divided each sample into 2 fractions: one was used to enrich ECs (CD45^–^, EPCAM^−^, CD31^+^, CD102^+^ cells) and the other one contained all stromal cells. We based the selection of ECs both on CD31 and CD102 to increase purity. For a representative overview of the FACS strategy for EC-enrichment, see Supplementary Fig. 10. For further processing for scRNA-seq, samples were resuspended in PBS containing 0.4% UltraPure BSA (50 mg/mL) (Thermo Fisher Scientific). Cells were counted using an automated cell counter (Luna FL, Logos Biosystems, Villeneuve d’Ascq, France) and processed as described below. Throughout the dissociation procedure, cells were maintained on ice. For details on antibodies (as well as their dilutions) and reagents used throughout this study, we refer to Supplementary Table 1.

### Single cell droplet-based RNA sequencing

The single cell suspensions were converted to separate barcoded scRNA-seq libraries using the Chromium Single Cell 3’ Library, Gel Bead & Multiplex Kit, and Chip Kit (10X Genomics, v2), aiming for 1500 cells per library. For each patient, all samples (TEC, pEC, TME, pME) were processed in parallel in the same thermal cycler. Libraries were sequenced on an Illumina HiSeq4000. The enriched EC samples of patient #6 were not sequenced due to technical reasons and the enriched pEC sample of patient #1 and pME sample of patient #2 were sequenced but excluded in the analysis after quality control.

### scRNA-seq cohort description and characteristics

Only hormone sensitive, early stage, treatment-naïve BC patients, who underwent resection (broad excision or mastectomy) were included for single cell RNA-sequencing and protein validation. Patient characteristics are listed in Supplementary Data 1. Informed written consents were obtained before surgery. TNM staging for BC was done according to the 8th edition of the IUCC guidelines.

### scRNA-seq data analysis

Gene expression matrices were generated using Cell Ranger (10X Genomics, version 2.1.1) using the GRCh38 build of the human reference genome, and further processed using R (version 3.4.4). We used the following quality control steps: genes expressed by <10 cells or with a row mean of <0.003 were not considered, cells expressing <300 genes (low quality), a number of genes >2 standard deviations above the mean (potential doublets), or >10% of unique molecular identifiers (UMIs) derived from the mitochondrial genome were removed. Visualization, clustering, marker gene identification, trajectory analysis and gene set enrichment analysis of the remaining cell data was performed using BIOMEX software^101^. Briefly, after normalization and auto-scaling, the resulting data were first summarized by principal component analysis (PCA), followed by visualization using Uniform Manifold Approximation and Projection (UMAP; n.neighbors = 10, minimal distance = 0.3), and graph-based clustering using Seurat. In case of enriched EC data, clusters containing contaminating immune cells (*PTPRC*^+^), epithelial cells (*KRT15*^+^), fibroblasts (*DCN*^+^, *LUM*^+^), smooth muscle cells (*ACTA2*^+^) and pericytes (*RGS5*^+^) were removed. Additionally, clusters not expressing the canonical EC marker genes *CDH5* and *PECAM1* were removed. To estimate the number of distinct phenotypes in our data, we color-coded UMAP-plots for each of the ~14,000 detected genes (Brute force, implemented in BIOMEX) and identified clusters of cells with discriminating gene expression patterns in all samples. Next, we applied graph-based clustering (using Seurat), using a clustering resolution that captured all expected clusters (verified by UMAP visualization (Supplementary Data 2).

For the micro-environment (non-enriched) samples, we followed a similar data processing pipeline as for ECs and obtained 18,082 cells. The first 40 PCs were visualized using UMAP (n.neighbors = 10, minimal distance = 0.3). We subclustered all major cell types (in clusters >100 cells) separately (for details see Supplementary Data 3). Despite our initial quality control, in some cell types we identified subclusters with a lower number of detected genes/cell as compared to the majority of subclusters. These subclusters did not contain strong and/or cluster-specific marker genes but expressed clear cell-type specific canonical marker genes. Therefore, instead of removing these subclusters, we refer to them as lower sequencing depth (LS). Moreover, within the immune cell compartment we identified a few subclusters likely representing doublets/low quality cells (358 cells in total), which were not used for further analyses (e.g., receptor–ligand interaction predictions).

Marker genes for each cluster were calculated by pairwise differential analysis of all clusters against all other clusters separately (using the “uniquely upregulated” function as implemented in the “marker set analysis” in BIOMEX, followed by ranking of the results of each pair-wise comparison by log2 fold change^102^). The most upregulated genes received the lowest rank number (top ranking marker genes). For each cluster, we combined the rank numbers for all genes in all pair-wise comparisons by calculating their product to obtain a final list of ranked marker genes for each cluster.

#### Bootstrap probability analysis

For hierarchical clustering, we applied Euclidean distance and complete linkage, and used the *pvclust* R package to estimate the confidence of each branch of the tree using the bootstrap resampling approach (number of bootstrapping = 1000; confidence score >0.4, as used in previous reports^19,103,104^. The following *pvclust*-calculated *p*-values for hierarchical clustering are also provided: (i) “approximately unbiased” (AU) *p*-values, computed by multiscale bootstrap resampling, and (ii) “bootstrap probability”(BP) values, computed by normal bootstrap resampling^27^. AU *p*-values are less biased compared to BP values, and clusters with AU ≥ 95% are strongly supported by the data^27^.

#### Pseudotime analysis

Analysis was performed as implemented in BIOMEX using SCORPIUS^105^. We included EC subclusters belonging to different traditional vascular beds that grouped together in the hierarchical clustering analysis as depicted in Fig. 1e: arteries (EC2–3), capillaries (EC11–12), and veins (EC8–10). Activated PCV (EC6; not considered a classical vein; Supplementary Data 3) and vein ii (EC7; patient-specific) were not included in the analysis, as well as the angiogenic ECs (angiogenic (EC4), angiogenic – LS (EC5)), and lymphatic (EC1) ECs. We used the highly variable genes (mean lower threshold 0.05; mean higher threshold 8) and the following parameters: *k* = 3, number of principal components = 8. For visualization, clusters detected in pseudotime were smoothened using quantiles (0.25 and 0.75 for lower and upper quartiles, respectively). Clusters were ordered based on their lower quantile value; new cluster value ranges were defined by averaging together the upper quantile of the first cluster and the lower quantile of the second cluster. The newly defined cluster ranges were used for plotting. Regression span = 0.2 (local linear regression).

#### Comparison of breast EC taxonomy with publicly available EC data

We used a scRNA-seq dataset of (peri-) tumoral human lung tissue^19^. Raw counts and the accompanying metadata were downloaded from https://www.vibcancer.be/software-tools/lungTumor_ECTax. Marker genes were calculated for every lung EC cluster (patient #5-specific cluster was excluded) as described for processing of the breast scRNA-seq dataset, using patient information as a covariate^19^. The *scmapCluster* algorithm, as implemented in BIOMEX (*scmap* package, version 1.1.5)^28^, using a list of all top-25 marker genes for each lung EC cluster and a similarity threshold of 0.5 (all other parameters were default) was used to assign the lung EC subclusters to the breast EC data. For congruency analysis, we calculated the similarity of marker genes (calculated using all expressed genes in both datasets) using pairwise Jaccard similarity coefficients for all clusters against all other clusters. The Jaccard coefficient is defined as the size of the intersection divided by the size of the union sets:

1 $$J\left(A,B\right)=\frac{{{{{{\rm{|}}}}}}A\cap B{{{{{\rm{|}}}}}}}{\left|A\cup B\right|}=\frac{{{{{{\rm{|}}}}}}A\cap B{{{{{\rm{|}}}}}}}{{{{{{\rm{|}}}}}}A{{{{{\rm{|}}}}}}+{{{{{\rm{|}}}}}}B{{{{{\rm{|}}}}}}-{{{{{\rm{|}}}}}}A\cap B{{{{{\rm{|}}}}}}}$$

where *J* is the Jaccard index and *A* and *B* are two sets of marker genes^106^.

For the comparison of our breast EC taxonomy to additional cancer types, we used a scRNA-seq dataset of human breast, ovarian lung and colorectal cancer^97^. Raw counts were downloaded from https://lambrechtslab.sites.vib.be/en/data-access, and ECs were subsetted based on provided cell type annotations. The *scmapCluster* algorithm was used as described above, using a list of all top-25 marker genes for each breast EC cluster and a similarity threshold of 0.5 (all other parameters were default) to assign the breast EC subclusters to the external EC data.

#### Gene set analyses

DGEA (differential gene expression analysis), GSEA (gene set enrichment analysis), and GSVA (gene set variation analysis)^107^ were performed as implemented in BIOMEX^101^. In short, we first performed pairwise differential gene expression analysis for each EC subcluster of interest *versus* all remaining EC subclusters, using the *limma* package^108^. The resulting list of DEGs was then filtered, selecting only those DEGs with an adjusted *p*-value < 0.05 (Benjamini–Hochberg).

An in-house curated list of 910 metabolism-related gene sets (Supplementary Data 2) obtained and selected from the Molecular Signatures Database was used for competitive GSEA and GSVA^72^. GSVA scores were calculated for sets with a minimum of 5 detected genes, all other parameters were default. For GO enrichment analysis, we ranked significant DEGs in each DGEA by fold change (the most upregulated gene received rank 1). The top 100 significant DEGs from this filtered list (sorted by log fold change) were then used for GO enrichment analysis using the *ClusterProfiler* package^107^, using a *p*-value cut-off of <0.01, and a *q*-value (Benjamini–Hochberg) cut-off of <0.05.

#### Receptor–ligand interaction analysis

To investigate the potential EC-stromal cell interactome in our data, we used the Python implementation of *CellPhoneDB*^45^. As input to the algorithm, we used a pooled normalized count matrix, comprising both the EC-enriched and (T)ME (no EC-enrichment) datasets, and the following parameters: data=hgnc_symbol; iterations=500; threshold=0.25; subsampling-log=false; subsampling-num-cells=9000. Only significant interactions (“significant_means” output file) were used for further analysis. EC-self interactions, or interactions between EC subclusters and mast cells, plasmacytoid dendritic cells, or plasma cells (all <100 cells per cluster) were excluded. EC subclusters identified in the (T)ME dataset (not EC-enriched) were also not considered for the analysis. To ensure EC subcluster specificity of the interaction, for each EC subcluster of interest (either angiogenic ECs (EC4) or venous ECs (EC7–10)) only receptors/ligands enriched at a log2fold change ≥0.25 (as compared to all other EC subclusters) were considered. Moreover, for predicted interactions between venous ECs and the stroma, the average interaction of all venous subclusters (EC7–10) was calculated, and only interactions occurring in at least 3 out of 4 venous subclusters were considered. Interactions were visualized as circos plots using the *circlize* R package^109^. While interactions were calculated between all identified subclusters, we specifically focused our interpretation and analysis on interactions between angiogenic or venous ECs and immune cell types (all NK, T, and myeloid subclusters).

#### NicheNet analysis

NicheNet analysis was performed using the *nichenetr* package^110^ (v. 1.0.0)) and the accompanying Seurat vignette (https://github.com/saeyslab/nichenetr/blob/master/vignettes/seurat_steps.md). We only considered genes expressed in at least 10% of the sender or receiver populations. The following subclusters were used in the analyses: angiogenic ECs (cluster EC4), all (pooled) myeloid subclusters (Mye1–7), or conventional DCs (cluster Mye6). The gene sets of interest (DEGs between tumor- and peri-tumor-derived cells) were identified using the *FindMarkers* function in Seurat (adjusted *p*-value ≤0.05, average log fold change ≥0.25). As described for the receptor-ligand analysis above, to ensure EC subcluster specificity of the interaction, for angiogenic ECs (EC4) the list of potential ligands was filtered to only include genes enriched at a log2fold change ≥0.25 (as compared to all other EC subclusters). Ligand activities were predicted using the *predict_ligand_activities* function, using the pearson correlation coefficient, and top-ranking ligands were selected for further analysis (based on histogram evaluation of ligand activity scores). Default settings were used for all other aspects of the analysis.

#### SCENIC analysis

Analysis was performed using the SCENIC R package^80^ with the enriched EC dataset, using the 20,000 motifs database for RcisTarget and GRNboost (SCENIC version 0.1.5, which corresponds to RcisTarget 0.99.0 and AUCell 0.99.5; with RcisTarget.hg19.motifDatabases.20k).

#### inferCNV analysis

CNVs (determined for luminal cell annotation, see Supplementary Information) were estimated using the *inferCNV* R package (version 1.5.0) as described in previous studies^111,112^. For this analysis, we only made use of the (T)ME (not EC-enriched) dataset, of which we selected all immune, fibroblast, myoepithelial, and (peri-)vascular cells as a normal (non-malignant) reference to estimate the presence of CNVs (indicative of malignant cells) in all luminal subclusters. Analysis was performed using the following parameters: cut-off 0.1, denoise = T, HMM = T, cluster_by_groups = T.

### Tissue staining and quantification

#### IHC staining

Formalin-fixed paraffin-embedded human breast tissue sections (thickness 7 µm) were subjected to hematoxylin/eosin staining or immunohistochemistry. For a full list of primary and secondary antibodies used for immunohistochemistry, see Supplementary Table 1. Briefly, after incubation overnight at room temperature with the primary antibodies, sections were incubated with the appropriate secondary antibodies followed by amplification with the proper tyramide signal amplification system (Perkin Elmer) (CD105, ACKR1, SMA, CD36, HLA-DRA, CLEC2B, CD16, and KLRF1) or with DAB Substrate Kit (Abcam) (CD3, CD68, CK18/8, and CK5/6) or with Alexa Fluor-conjugated secondary antibodies (FABP4, INSR) or with Akoya’s Opal™ Multiplex IHC system (CD105, INSR, SELL, PODXL, and FOXP3). Nuclei were counterstained with Hoechst 33342 (Sigma-Aldrich) and slides were mounted using ProLong Gold Antifade Mounting medium (Thermo Fisher Scientific). Imaging was performed using a Zeiss AxioScan Z1 at 20× magnification, or by confocal imaging using a Zeiss LSM780 confocal microscope (Carl Zeiss) at 100× magnification (alpha Plan-Apochromat 100×/1.46 Oil DIC M27). The images were processed using Fiji software (https://fiji.sc).

#### RNAscope in situ hybridization

Formalin-fixed paraffin-embedded human breast tissue sections were subjected to RNAscope in situ hybridization using the RNAscope Multiplex Fluorescent v2 assay (ACDBio) combined with immunofluorescence—Integrated Co-Detection Workflow according to the manufacturer’s instructions (Pretreatment and RNAscope Multiplex Fluorescent v2 Assay according to protocol 323100-USM and MK-5150). Hybridization was performed with the RNAscope probes (ACKR1, FABP4, PPARG, ID2), and the RNAscope 3-plex Positive and Negative Control Probes (see Supplementary Table 1). Slides were then processed according to the RNAscope Multiplex Fluorescent v2 protocol (Hybridization, Amplification, and Signal Development), combined with immunofluorescence for CD105 or CD105 and HLA-DR and counterstained with Hoechst 33342 (Sigma-Aldrich). Images were acquired using a Zeiss LSM 780 confocal microscope (Carl Zeiss).

#### Quantification

For quantification of IHC or H&E stainings, we quantified one complete tissue section per patient per condition. For quantification of images containing RNAscope probes, we analyzed 10 representative images per patient per condition. For the quantification of HLA-DR expression in ACKR1^+^ vessels, we regarded a blood vessel as ACKR1^+^ if a clear pattern on RNAscope in situ hybridization was present (Fig. 2f). To compare PPAR-γ expression in LIPECs *versus* non-LIPECs we quantified the % of *PPARG*^+^ nuclei in CD105^+^, *FABP4*^+^ blood vessels, or CD105^+^, *FABP4*^–^ blood vessels, respectively, in the same patient. For the validation of the major cell types (Supplementary Fig. 4e–g) and the quantification of LIPECs (Fig. 5f, g), we used tissue sections from the same patients as used for scRNA-seq. We used specific staining for luminal cells (CK8/18, a mixture of normal luminal cells and BC cells), myoepithelial cells (CK5/6), ECs (CD105), T cells (CD3), and macrophages (CD68) to quantify their abundances. An expert breast pathologist from the University Hospital Leuven quantified perivascular cells and other stromal cells (a mixture of fibroblasts and adipocytes) on H&E images. For quantification of FAPB4^+^ vessels (LIPECs) we quantified the CD105^+^, FABP4^+^ area relative to the CD105^+^ area. For morphometric analysis, we used Leica MetaMorph AF 1.8 software package.

### HUVEC and NK cell co-cultures

Human umbilical vein endothelial cells (HUVECs) were freshly isolated from umbilical cords obtained from multiple donors of unknown sex (with approval from the Ethics Committee Research UZ/KU Leuven and informed written consent obtained from all subjects) as previously described^113^. Briefly, the interior of the umbilical vein was rinsed with PBS containing antibiotic-antimycotic solution (Thermo Fisher Scientific) and injected with pre-heated collagenase I solution (0.2% collagenase type I in 0.9% NaCl, 2 mM CaCl2, antibiotic-antimycotic). After no more than 13 min incubation, the collagenase suspension containing endothelial cells (ECs) was collected, filtered through a 40 μm nylon cell strainer, and spun down. The ECs were plated on 0.1% gelatin-coated dishes in M199 medium (1 mg/mL D-glucose) (Thermo Fisher Scientific) supplemented with 20% fetal bovine serum (FBS) (Merck-Biochrom), 2 mM L-glutamine (Thermo Fisher Scientific), Endothelial Cell Growth Supplement (ECGS)/ Heparin (PromoCell), 100 IU/mL penicillin and 100 μg/mL streptomycin (Thermo Fisher Scientific), and cultured until confluent in a 5% CO_2_, 37 °C incubator. The confluent cultures were split and replated in a 1:1 mixture of M199 and endothelial cell basal medium (EGM2) (PromoCell) supplemented with endothelial cell growth medium supplement pack (PromoCell) and further cultured in EGM2 medium. In all experiments, HUVECs were used as single-donor cultures and were used between passages 2 and 5. Cultures were regularly tested for mycoplasma. HUVECs were transduced in 10 cm^2^ petri-dishes with 15 MOI lentiviral vectors containing empty control (PLKO) or two different shRNA constructs for CLEC2B knockdown (KD). After 24 h the medium was refreshed. 4 days after transduction, HUVECs were trypsinized, replated in gelatin-coated 48-wells plates and cultured for 24 h with PBS (control) or 2 μg/mL lipopolysaccharide (LPS, Sigma) and 100 ng/mL interferon gamma (IFNγ, Peprotech) to induce CLEC2B expression.

For natural killer (NK) cell isolation, whole blood was acquired from a healthy volunteer in heparin coated tubes. Then, PBMCs were isolated from whole blood by Ficoll-Paque (GE Healthcare) followed by density centrifugation, after which the PBMC fraction was collected in PBS and washed 2 times with PBS. NK cells were isolated by negative isolation using the NK cell isolation kit (Miltenyi Biotech) as per the manufacturer’s instructions. Isolated NK cells were washed and resuspended in RPMI supplemented with 10% heat-inactivated FBS. Then, HUVECs were washed twice with RPMI medium and 100,000 NK cells/well were added to the confluent HUVEC monolayer in each well. The co-culture was incubated for 24 h. After the co-culture, cells were harvested and supernatant was collected and frozen at −20 °C until use. The cells were washed with FACS buffer (PBS + 0.5% BSA (Sigma) and 2 mM EDTA) and stained for the following markers: CD45 (BV421), CD56 (BV650), CD107a (FITC), CD3 (AF700) and viability dye (eFluor 780 from eBioscience). All antibodies were purchased from Biolegend. FACS staining was performed for 30 min at 4 °C in the dark. After staining, cells were washed twice with FACS buffer, and samples were analyzed on a FACS Aria III (BD Biosciences). Data was analyzed using FlowJo software (v10.8.1; BD Biosciences). NK cells were gated as live, single CD45^+^CD3^−^CD56^+^ cells. The purity of NK cells was >95% of all CD45^+^CD3^−^ cells in each well and CD107a expression was assessed.

### RNA Isolation and quantitative RT-PCR

RNA was collected and purified with the PureLink RNA Mini Kit (Thermo Fisher Scientific) and converted to cDNA using the iScript cDNA synthesis kit (Bio-Rad). RNA expression analysis was performed with TaqMan Fast Universal PCR Master Mix (Thermo Fisher Scientific) as described using premade primer sets (IDT Integrated DNA Technologies). For comparison of gene expression between conditions, mRNA levels normalized to the housekeeping gene *HPRT*.

### Protein extraction and immunoblotting

Protein extraction and immunoblot analysis were performed using RIPA Lysis and Extraction Buffer (Thermo Fisher Scientific) in the presence of protease and phosphatase inhibitors (Roche). Lysates were separated by SDS-PAGE under reducing conditions, transferred to a nitrocellulose or PVDF membrane, and analyzed by immunoblotting. Primary antibodies and appropriate secondary antibodies are listed in Supplementary Table 1. Signal was detected using the ECL or Femto system (Thermo Fisher Scientific) according to the manufacturer’s instructions. Densitometric quantifications of bands were done with Fiji software. For calculation of the % reduction in CLEC2B expression relative to CTRL (empty control, PLKO), the CLEC2B/housekeeping gene ratio in the CLEC2B^KD^ conditions was determined separately for every HUVEC donor analyzed, and normalized to the CLEC2B/housekeeping gene ratio in the CTRL condition (in the same donor). alpha-Tubulin (*n* = 1) or GAPDH (*n* = 3) were used as housekeeping genes. See Supplementary Information for uncropped scans of the western blot shown in Supplementary Fig. 8c.

### Analysis of the retrospective clinical cohort

Patient data: All patient data used for retrospective clinical cohort analyses (and their validation) were retrieved from the multidisciplinary BC clinic database in the University Hospital Leuven. This study was approved by the Medical Ethics Committee UZ Leuven under protocol number S63779.

Patient demographics: We analyzed metadata of all 4924 female patients with early, hormone sensitive, HER2 negative BC treated with definitive surgery between 1987 and 2010 from the BC clinic database indicated above. All patients had a minimal follow up of 8 years. 265 patients were not considered for downstream analysis due to lack of critical metadata that could influence the survival analysis. Patients were compared by groups during BC follow up: (i) patients not taking metformin (or any other PPAR-γ agonist) and (ii) patients taking metformin (and thiazolinedione (1 patient)). We considered a patient in the metformin group only after continuous metformin intake of >1 year during follow up for BC, 11 patients discontinued metformin treatment before this cutoff and were excluded from the analysis. Group 1 contained 4290 patients (92.2%) who were not treated with metformin, while group 2 contained 358 patients (7.8%) who were treated with metformin in the context of diabetes mellitus. Patients in group 2 had more adverse clinical characteristics (they were older, had a higher Body Mass Index (BMI, a known risk factor for adverse outcomes in BC^114,115^) and had a larger fraction of patients with cancer stages II and III).

#### Survival analysis

We retrospectively analyzed the effect of metformin treatment on the clinical outcome in a cohort of 4648 female patients with early, hormone receptor-positive/HER2-negative BC, who underwent surgery followed by anti-hormonal treatment. Our cohort includes BC patients treated with the biguanide metformin (all diabetic), control patients that are diabetic but not treated with metformin, as well as non-diabetic controls without treatment (to increase statistical power). 357 out of 358 patients in the treatment group received metformin and the remaining 1 patient received both metformin and thiazolidinedione treatment. We used χ2 tests of independence to examine differences in the baseline characteristics between groups (Supplementary Data 6). We investigated the BCSS and DRFI between the two groups. Cumulative incidence function estimates of each group were compared using log-rank tests to estimate BCSS and DRFI. Death of other causes was considered a competing event. A group by time interaction was modeled to test the proportional hazards (PH) assumption. BCSS and DRFI were significantly better for the patients treated with metformin if the test for proportional hazards did not reject the assumption. In case this assumption is rejected (*p*-value < 0.05), the group-effect is estimated at two distinct follow-up times (5 and 8 years). All survival analyses were tested in a Cox proportional hazards regression model that was adjusted for: (i) body mass index (<30 *versus* ≥30), (ii) pathological stage (Stage I–II *versus* Stage III), (iii) age (age ≤ 50 years *versus* age > 50 years), (iv) tumor histological grade (G = I–II *versus* G > II) and (v) central hormone receptor status (double positive *versus* single positive). Because (i) standard of care adjuvant treatment (radiotherapy, chemotherapy, endocrine therapy) changed considerably during the recruitment interval and (ii) because its indications are mainly driven by the pathological stage, age, tumor histological grade, and central hormone receptor status, we did not perform a separate adjustment for adjuvant treatment modalities. The time to event or censoring was computed in years since diagnosis for each patient. Survival time was censored at the date of the last follow-up during the follow-up period if events were not observed. Survival probabilities and associated confidence intervals were estimated non-parametrically using the Kaplan–Meier product limit method at 5, 10, and 14 years. For cases with unknown cause of death (*n* = 286 on a total of 1350 deaths) we assumed disease-related death in presence of relapse (*n* = 72), and death of other causes in absence of relapse (*n* = 214).

#### Breast cancer outcomes based on individual matching

Each BC patient treated with metformin was compared to two control patients (non-diabetic BC patient not treated with metformin) matched for age, BMI, pathological stage, and histological grade. For one patient we could only assign one control. Survival analysis was done as described above. Here, we used Cox proportional hazard models for data analysis, accounting for clustering due to matching by using the robust sandwich estimate of Lin and Wei (1989)^116^. Results are presented as hazard ratios with 95% confidence intervals (Supplementary Data 6). Analyses were performed using version 9.4 of the SAS system for Windows.

### General statistics

Data are represented as mean ± SEM. When comparing two groups for a single parameter a paired t-test was used, while for three groups a one-way ANOVA followed by Dunnett’s multiple comparisons tests was used (in GraphPad Prism8).

### Reporting summary

Further information on research design is available in the Nature Research Reporting Summary linked to this article.

## Supplementary information

Supplementary Information

Description of Additional Supplementary Files

Supplementary Data 1

Supplementary Data 2

Supplementary Data 3

Supplementary Data 4

Supplementary Data 5

Supplementary Data 6

Reporting Summary

## Data Availability

The raw and processed single cell RNA sequencing data generated in this study have been deposited in the gene expression omnibus database (GEO), under accession code GSE155109. The publicly available lung cancer EC data used in this study are available in the ArrayExpress database at EMBL-EBI under accession code E-MTAB-6308, and at https://carmelietlab.sites.vib.be/en/software-tools (lung Tumor ECTax)^19^. The publicly available breast, ovarian and colorectal cancer data used in this study are available in the ArrayExpress database at EMBL-EBI under accession code E-MTAB-8107, and at https://lambrechtslab.sites.vib.be/en/data-access^97^. Source data are provided with this paper. The remaining data are available within the Article, Supplementary Information or Source Data file.
