# Supplementary material for: Single cell atlas identifies lipid-processing and immunomodulatory endothelial cells in healthy and malignant breast
Source: Nat Commun. 2022 Sep 20;13:5511. doi: 10.1038/s41467-022-33052-y (PMC9489707; doi:10.1038/s41467-022-33052-y)
Supplement: Supplementary file 1 — Supplementary Information [file 41467_2022_33052_MOESM1_ESM.pdf]

Single cell atlas identifies lipid-processing and immunomodulatory endothelial cells in healthy and malignant breast

Supplementary information

Annotation of luminal and immune cell subclusters

Luminal subcluster annotation – nomenclature was based on a combination of sample origin and gene expression signatures. Clusters L1-5 highly expressed genes involved in hormone receptor signaling (*ESR1, PGR, AR, TFF1, ANKRD30A*), cell proliferation (*TOP2A, BIRC5*), and/or known breast cancer (BC) genes (*GATA3* and *MUC1*)[1,2] (Supplementary Fig. 5a), suggestive of their malignant nature. Interestingly, subcluster L5 showed a gene expression signature resembling malignant epithelial cells in our dataset but was predominantly derived from peri-tumoral mammary tissue (Supplementary Fig. 5a-d), raising the question of whether this subcluster may consist of breast cancer cells that invaded the surrounding peri-tumoral tissue. Luminal subclusters L6-8, on the other hand, expressed genes known to be expressed in secretory and mature 'healthy' luminal cells (*SLPI, ELF5, KIT*)[3,4], with a low or absent expression of hormone receptor signaling (Supplementary Fig. 5a). Cluster L6 was predominantly derived from the tumoral tissue (Supplementary Fig. 5c) but closely resembled the secretory and mature luminal phenotype (Supplementary Fig. 5a), indicating that non-cancerous 'healthy' luminal cells may exist within the TME. This finding is also observed by other groups, but with unclear biological relevance[5].

We further explored these findings by inferring chromosomal copy number variations (CNVs) from the gene-expression data using the inferCNV algorithm, as described in the previous studies[6,7]. We selected all immune, fibroblast, myoepithelial and (peri-)vascular cells detected in our dataset as a normal (non-malignant) reference to estimate the presence of CNVs (indicative of malignant cells) in all luminal subclusters. This analysis revealed a dominant gain of the long (q) arm of chromosome 1 in clusters L1 and L2, largely derived from tumoral tissues (Supplementary Fig. 5d). Previous work indeed identified gain of 1q as the most frequent chromosomal arm-level event in gynecologic and breast cancers, occurring in almost 50% of analyzed samples[8], suggesting that the L1 and L2 subclusters in our dataset are indeed of malignant nature. Other frequently observed CNVs are gain of 8q and loss of 13q[8], both of which we observed in clusters L3 and L4 (derived largely from tumoral tissue) (Supplementary Fig. 5b,c) again suggesting their likely cancerous phenotype.

Clusters L6-8 did not share these CNVs and instead largely resembled the reference cells, suggestive of their non-malignant nature, and in line with their predominant peri-tumoral origin (Supplementary Fig. 5b,c). The only exception was an observed gain of 11p, uniquely detected in cells derived from peri-tumoral tissues. This particular CNV has also been observed in non-malignant epithelial cells in other studies profiling breast cancer tissue CNVs[9,10], and is thus unlikely to reflect a malignant nature of these cells.

Lastly, approximately half of cluster L5 presented with a mixture of CNVs resembling clusters L1-4, whereas the remaining cells resembled clusters L6-8, indeed suggesting this cluster may harbor cancerous cells invading peri-tumoral tissue, as described above.

Immune subcluster annotation – clusters of myeloid or lymphoid immune cells were annotated using canonical marker genes of major immune cell populations and subpopulations, marker genes from other studies employing scRNA-seq on (breast) tumor immune cells, and other available literature[11-20]. In brief, major myeloid subpopulations in the myeloid cell subclusters, containing macrophages, neutrophils, and conventional dendritic

cells, were identified by examining the expression of *CD14*, *CD68*, *C1QA* (macrophages); *S100A8, S100A9, CXCL2* (neutrophils) and HLA-genes, *CD1C*, *CCR7* (dendritic cells). Subpopulations of macrophages were annotated using literature and gene sets for tissue-resident-like, TAM, M1- and M2-like macrophages from[15,16,21-23]. The presence of subtypes of conventional dendritic cells or neutrophils was assessed using literature[18,24].

Even though NK cells can originate from lymphoid and myeloid precursors, NK cells and T cells were subclustered together as performed by others[25]. $\alpha\beta$T cells were identified by *CD3D*, *CD3E*, *TRAC*, *TRBC2,* and absence of *TRD*/*TRC* genes. *CD4* and *CD8A* expression was used to annotate $CD4^+$ or $CD8^+$ T cells. $CD4^+$ T cell subclusters were identified by assessing expression of markers indicating a naïve/activated/memory phenotype (e.g. *IL2RA*, *IL7R*, *CD44*, *CD69*, *CCR7*, *SELL*), regulatory T cells (e.g. *IL2RA*, *TIGIT*, *FOXP3*), T helper cell genes (Th1/Th2/Th17 canonical transcription factors/cytokines) and using scRNA-seq literature describing T cell heterogeneity in breast cancer[12]. Similarly, $CD8^+$ T cell subclusters were annotated by assessing expression of markers indicating a naïve/memory phenotype (*IL2RA*/*CD44*/*CD69*/*CCR7*/*SELL*), effector phenotype (cytotoxic T cell markers, such as *GZMB*, *PRF1*, *IFNG*, *TNF*), or markers whose high expression is associated with an exhausted state (e.g. *PDCD1*, *HAVCR2*, *LAG3*). Tissue-resident memory T cells were identified using literature[26,27]. and by assessing the expression of *ZNF683*, *PRDM1*, *ITGA1*, *CXCR6*, *ITGAE*, *CD69* and downregulated expression of *CD28*[27]. NK cell subclusters, lacking T cell genes, were annotated based on the expression of *NKG7*, *KLRB1*, *KLRF1*, *NCAM1,* and *FCGR3A* and gene expression of cytolytic effector molecules such as *GZMB* and *PRF1*, chemokine expression (*XCL1, XCL2*) and using a cross-species NK cell taxonomy[14].

Supplementary Figures

Supplementary Fig. 1

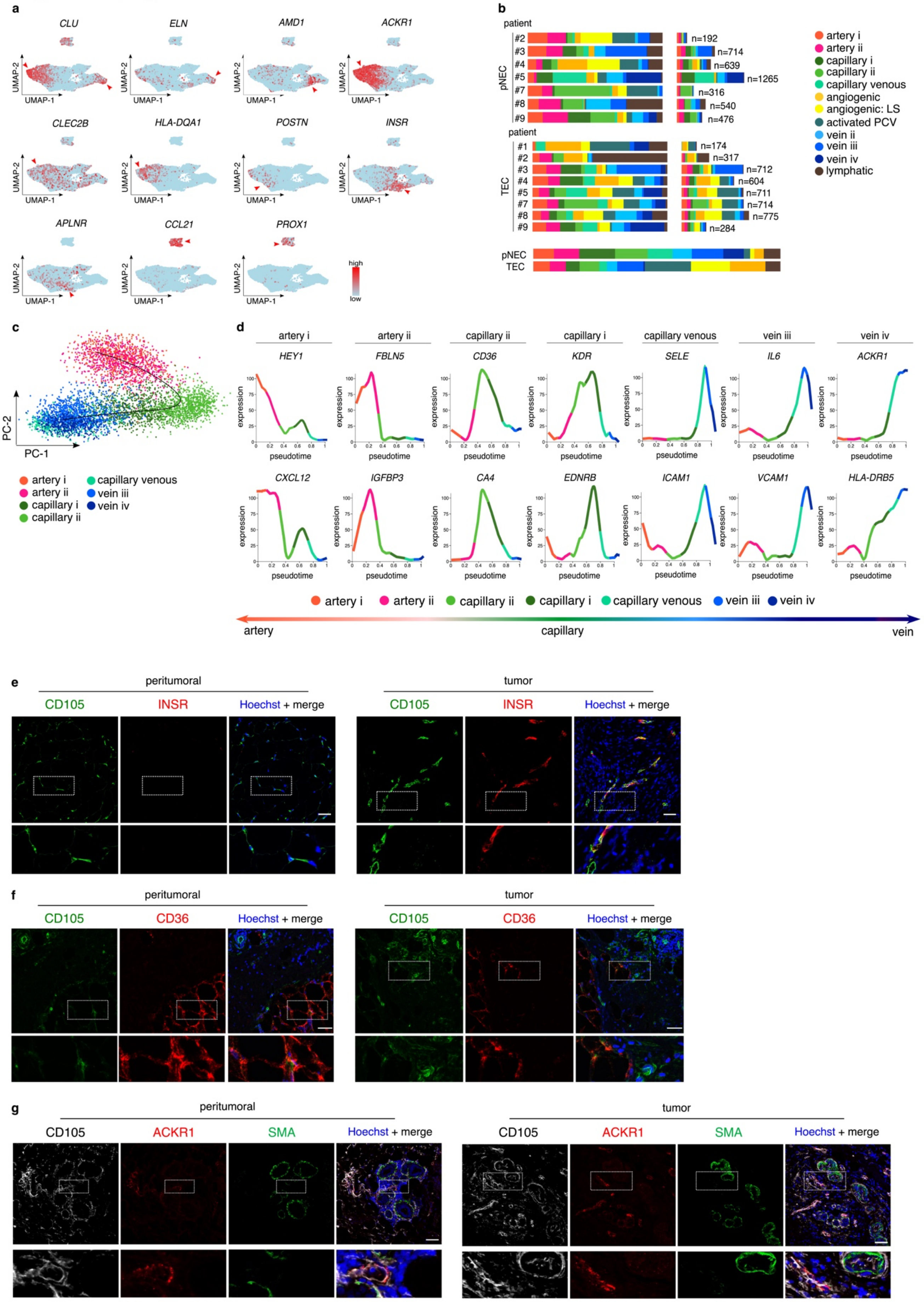

Supplementary Fig. 1: Single cell taxonomy of endothelial cells in the breast. a UMAP-plots, color-coded for the expression of representative marker genes in EC subclusters. Red arrows indicate cells highly expressing the marker gene. Color scale: red – high expression, blue – low expression. **b** Composition of EC subtypes in peri-tumoral (pEC) and tumoral (TEC) breast samples per patient. To correct for differences in absolute numbers of tumoral *versus* peri-tumoral cells, numbers in each subcluster were divided by the total number of tumoral and peri-tumoral cells, respectively (relative representation). Left: Relative contribution of each phenotype scaled to 100%. Right: Contribution of each phenotype in absolute numbers; the total number of analyzed ECs on the right. Bottom panel: relative contribution of ECs per condition, color coded per subcluster. EC – endothelial cell, LS – lower sequencing depth, PCV – post-capillary venule. **c** Pseudotime trajectory of arteries, capillaries, and veins, color coded according to cluster origin. PC – principal component. **d** Pseudotime analysis represented by loess regression-smoothened gene expression of indicated marker genes of arteries, capillaries, and veins, color coded according to subcluster. **e** Representative micrographs of human breast peri-tumoral (top) and tumoral (bottom) tissue sections, immunostained for CD105, INSR and counterstained with Hoechst (n=3). Lower panels: magnifications of the boxed areas in the upper panels. Scale bar: 50 µm. **f** Representative micrographs of human breast peri-tumoral (top) and tumoral (bottom) tissue sections, immunostained for CD105, CD36, and counterstained with Hoechst (n=3). Lower panels: magnifications of the boxed areas in the upper panels. Scale bar: 50 µm. **g** Representative micrographs of human breast peri-tumoral (top) and tumoral (bottom) tissue sections, immunostained for CD105, ACKR1, smooth muscle actin (SMA) and counterstained with Hoechst (n=3). ACKR1 is detectable in thin-walled SMA-negative (presumably) veins/venules. Lower panels: magnifications of the boxed areas in the upper panels. Scale bar: 50 µm.

Supplementary Fig. 2

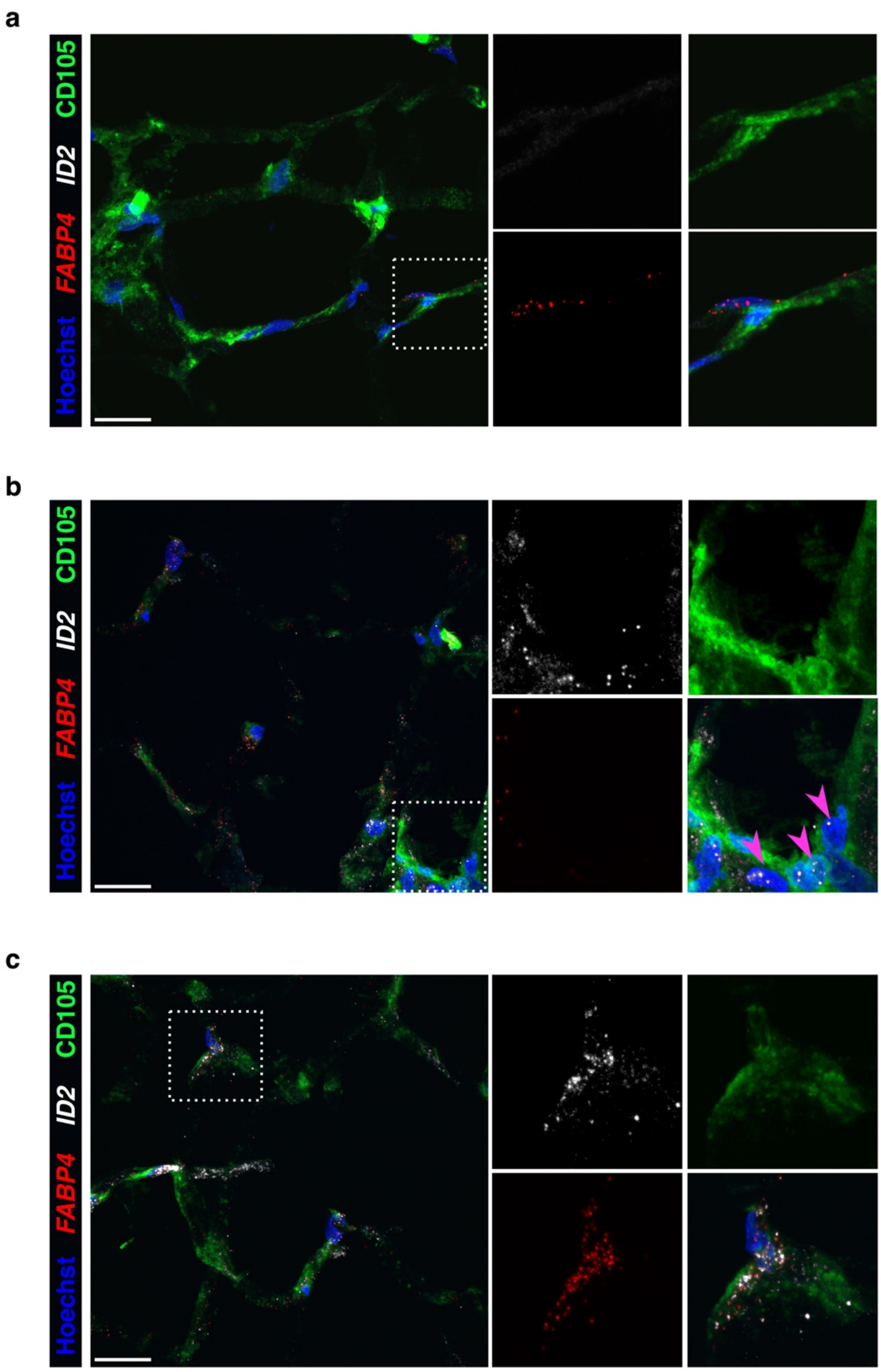

Supplementary Fig. 2: Expression of microvascular mutually exclusive markers. a-c Spatially restricted expression of *ID2* and *FABP4*, top-ranking marker genes of capillary i (EC11) and capillary ii (EC12) subclusters, respectively. Representative images of a human breast tissue section are shown, immunostained for CD105 (green) and counterstained with Hoechst (blue). Microvascular ECs exclusively expressing *FABP4* (a; red, RNAscope) or *ID2* (b; white, RNAscope), or expressing both *FABP4* and *ID2* (c) are shown. Right panels: magnifications of the boxed areas in the left panels. Arrows point at *ID2*$^{+}$*FABP4*$^{-}$ ECs. Scale bar: 25 µm, n=8.

Supplementary Fig. 3

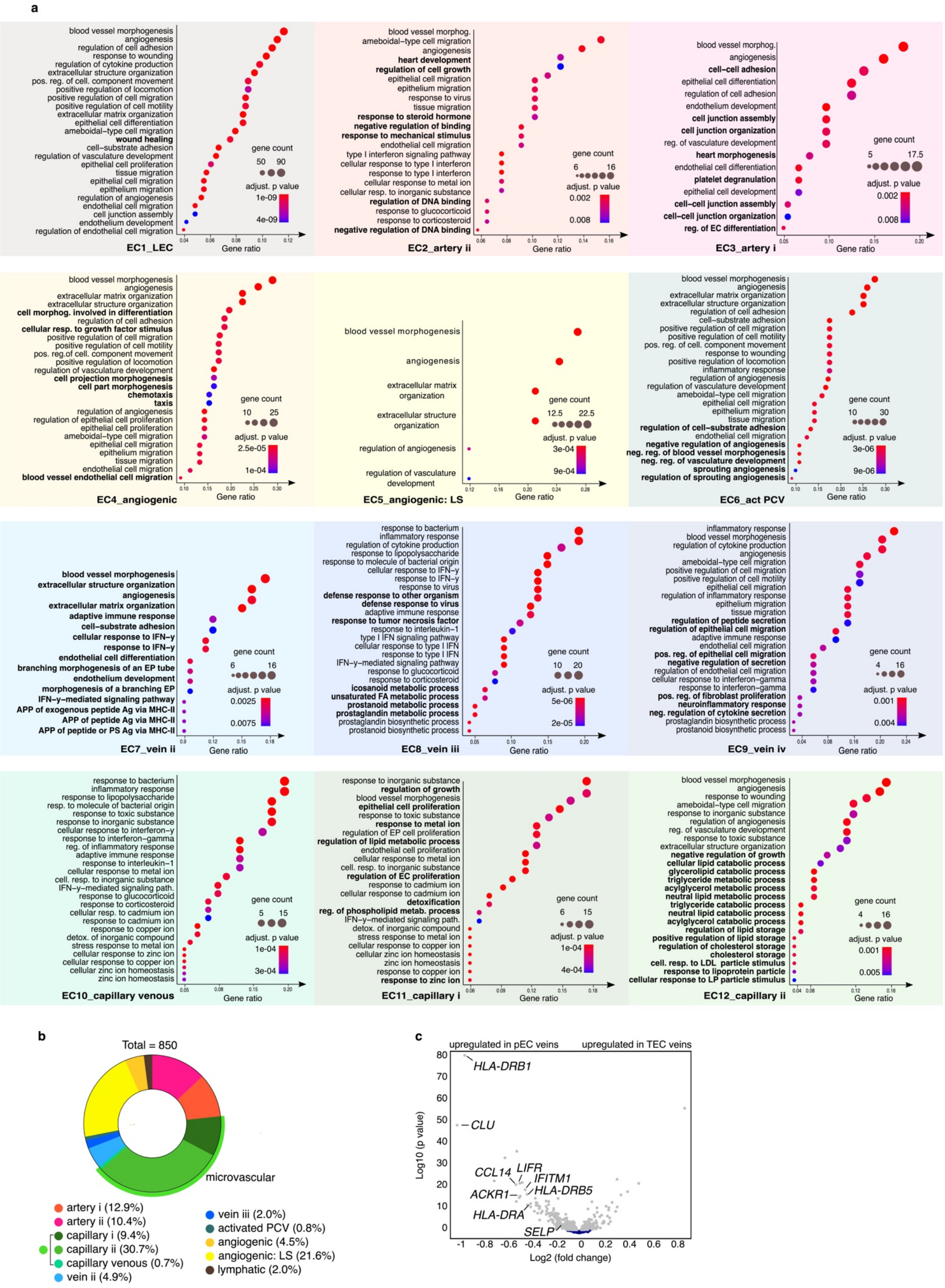

Supplementary Fig. 3: EC transcriptomic heterogeneity & congruency. a Dot plot of top-25 most upregulated pathways per EC subcluster, as calculated by GO enrichment analysis (ClusterProfiler), using a p-value cut-off of < 0.01, and a q-value (Benjamini-Hochberg) cut-off of < 0.05. Color scale indicates adjusted p-value; dot size depicts the number of enriched genes within each GO term; GO terms associated with distinct clusters are shown in bold. Abbreviations: Ag – antigen, APP – antigen processing and presentation, cell. – cellular, detox. – detoxification, EC – endothelial cell, EP – epithelial, FA – fatty acid, IFN – interferon, LDL – low-density lipoprotein, LP – lipoprotein, LS – lower sequencing depth, MHC-II – MHC class II, morphog. – morphogenesis, neg. – negative, PCV – post-capillary venule, pos. – positive, PS – polysaccharide, reg. – regulation, resp. – response. **b** Subcluster identity of breast ECs (n=850) that were unassigned by the scmap projection in Fig. 2a. LS – lower sequencing depth. **c** Volcano plot showing differential gene expression analysis of pEC veins *versus* TEC veins. Key pEC-enriched marker genes involved in immunoregulation are indicated. Gray, significant (adjusted p-value < 0.05); dark blue, not significant. Differential expression analysis was performed using *limma*, the magnitude of differential expression (log2 fold change) and false discovery rate adjusted p-values (calculated with the Benjamini-Hochberg method) are provided on the x- and y-axis, respectively.

Supplementary Fig. 4

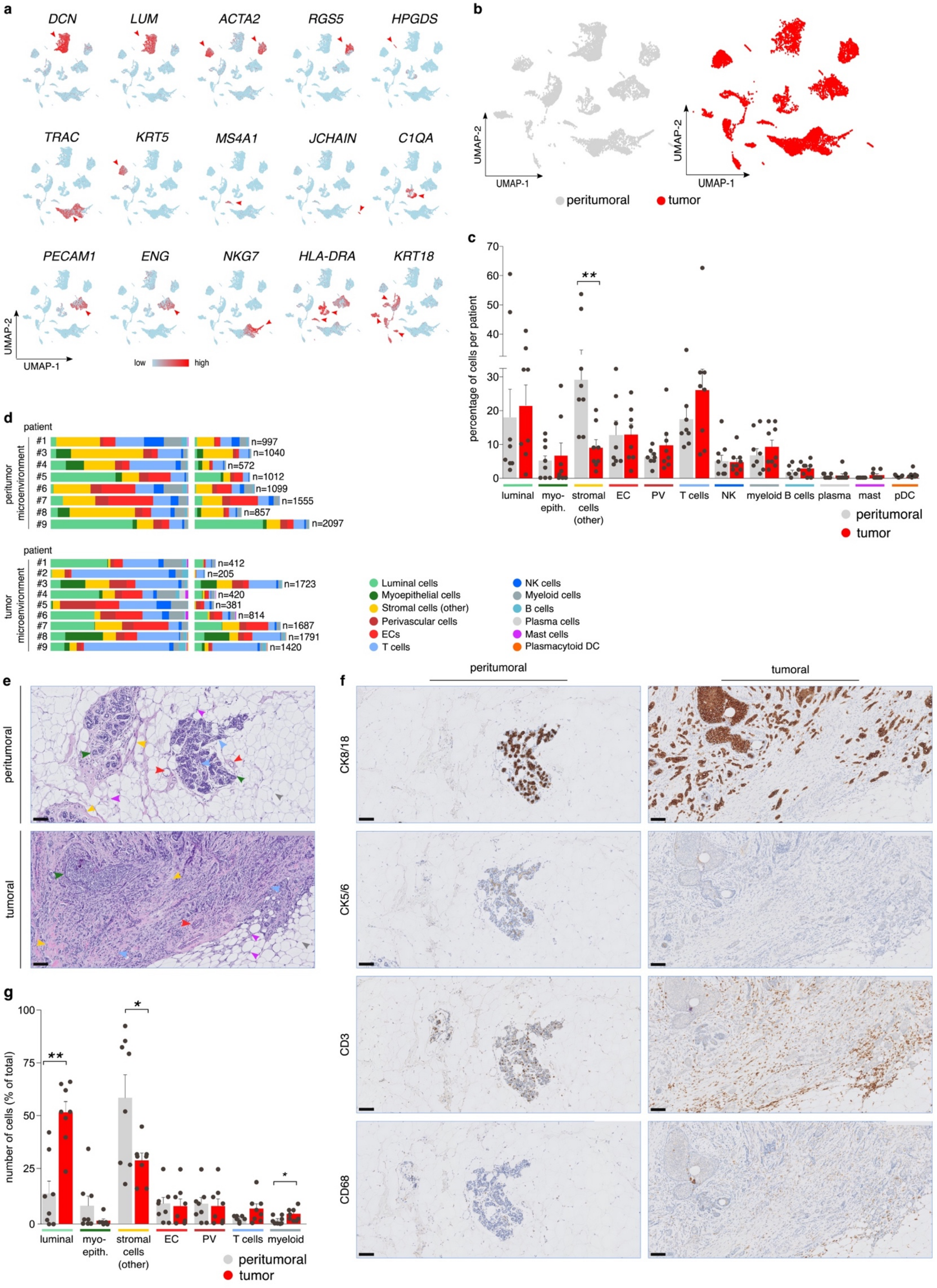

Supplementary Fig. 4: The breast microenvironment. a UMAP-plots of representative marker genes in the different major cell types. Red arrowheads indicate cells highly expressing the marker gene. Color scale: red – high expression, blue – low expression. **b** UMAP-plots of all 18,082 cells, color-coded by condition (peri-tumor (gray), tumor (red)). **c** Abundances of major cell types across conditions (peri-tumor (gray), tumor (red)), x-axis depicts major cell types color coded as in Fig. 3c. pDC – plasmacytoid dendritic cells, PV – perivascular, NK – natural killer. Data are mean ± SEM, n=8 for peri-tumoral samples and n=9 for TME samples, **$p<0.01$ (exact p-value=0.0053), a separate paired t-test (two-tailed) per major cell type (considering the 8 complete pairs). **d** Composition of major cell types in peri-tumoral (top panel) and tumoral (bottom panel) breast samples from individual patients. Left: Relative contribution of each phenotype scaled to 100%. Right: Contribution of each phenotype in absolute numbers; the total number of analyzed cells on the right. EC – endothelial cell, NK – natural killer, DC – dendritic cell. **e** Representative micrographs of human breast peri-tumoral and tumoral tissue sections, stained with hematoxylin and eosin (n=8). Scale bar: 100 µm. Arrowheads indicate cell types (blue, lymphocytes; purple, myeloid cells; red, blood vessels; green, myoepithelial cells; yellow, fibroblasts; gray, adipocytes). **f** Representative micrographs of human breast peri-tumoral and tumoral tissue sections, immunostained for breast epithelium (CK8/18), myoepithelial cells (CK5/6), T cells (CD3), and macrophages (CD68). Scale bar: 100 µm (n=8). **g** Abundances of major cell types in peri-tumoral and tumor tissue from BC patients, as quantified on H&E and immunostained sections; color coded per condition. Data are mean ± SEM, n=8, *$p<0.05$, **$p<0.01$ (exact p-values= 0.0029, 0.0335 and 0.0228, respectively), separate paired t-test (two-tailed) per cell type.

Supplementary Figure 5

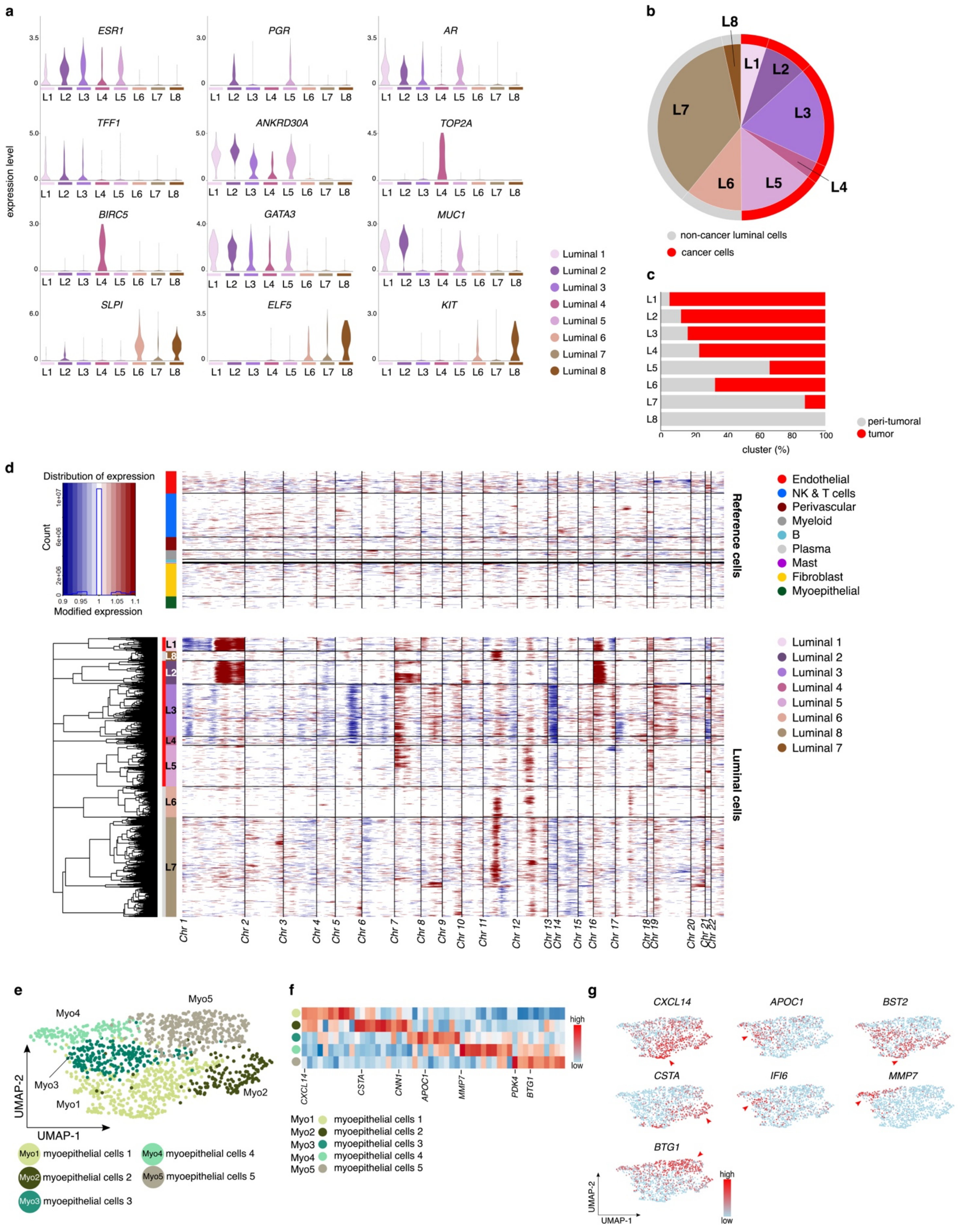

Supplementary Fig. 5: transcriptomic heterogeneity in the breast: epithelial cells. **a** Violin plots of the expression level of the indicated genes involved in hormone receptor and signaling pathways (*ESR1, PGR, AR, TFF1, ANKRD30A*); proliferation (*TOP2A, BIRC5*), BC markers (*GATA3, MUC1*), and breast maturation (*SLPI, ELF5, KIT*) in luminal subclusters. **b** Pie chart showing the sample origin (tumor- or peritumor) of luminal subclusters. Annotation is based on expression profile as indicated in panel a. **c** Composition of luminal subclusters in peri-tumoral and tumoral breast samples per patient. **d** Copy number profiles estimated from the scRNA-seq data (inferCNV analysis). Columns correspond to genes, ordered by chromosome position, rows correspond to cells. Top heatmap: reference cells, used to define baseline expression; all immune, fibroblast, myoepithelial and (peri-)vascular cells detected in our dataset were selected as a normal (non-malignant) reference. Bottom heatmap: reference cell expression data (top heatmap) is subtracted from the luminal cell expression data to yield differential/residual expression values; red indicates chromosomal region amplification; blue indicates chromosomal region deletion. Cells are clustered by luminal subcluster. **e** UMAP-plot of myoepithelial cells, color-coded by subcluster. **f** Heatmap of the expression levels of the top 10 marker genes in myoepithelial cell subclusters. Color scale: red – high expression, blue – low expression. **g** UMAP-plots of representative marker genes in the different myoepithelial cell subclusters. Red arrowheads indicate cells highly expressing the marker gene. Color scale: red – high expression, blue – low expression. Abbreviations: NK – natural killer, Myo – myoepithelial.

Supplementary Figure 6

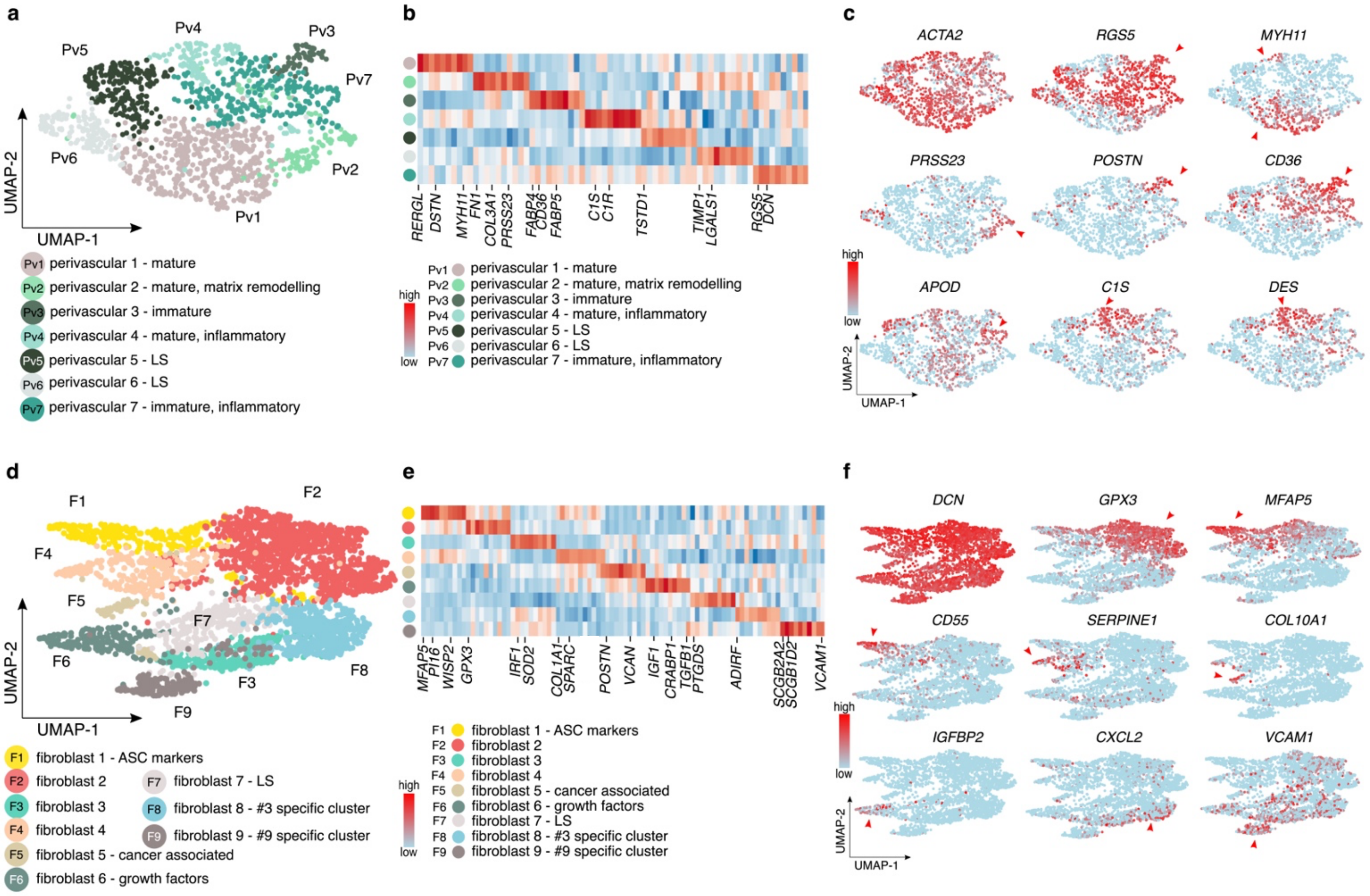

Supplementary Fig. 6: transcriptomic heterogeneity: perivascular and other stromal cells. a UMAP-plot of perivascular stromal cells, color-coded by subcluster. LS – lower sequencing depth. **b** Heatmap of the expression levels of the top 10 marker genes in perivascular stromal cell subclusters. Color scale: red – high expression, blue – low expression. LS – lower sequencing depth, PV – perivascular. **c** UMAP-plots of representative marker genes in the different subclusters in perivascular stromal cells. Red arrowheads indicate cells highly expressing the marker gene. Color scale: red – high expression, blue – low expression. **d** UMAP-plot of other stromal cells annotated as fibroblasts, color-coded by subcluster. ASC – adipocyte stem cell, LS – lower sequencing depth. **e** Heatmap of the expression levels of the top 10 marker genes in fibroblast subclusters. Color scale: red – high expression, blue – low expression. **f** UMAP-plots, showing expression of representative marker genes in the different subclusters of other stromal cells. Red arrowheads indicate cells highly expressing the marker gene. Color scale: red – high expression, blue – low expression.

Supplementary Fig. 7

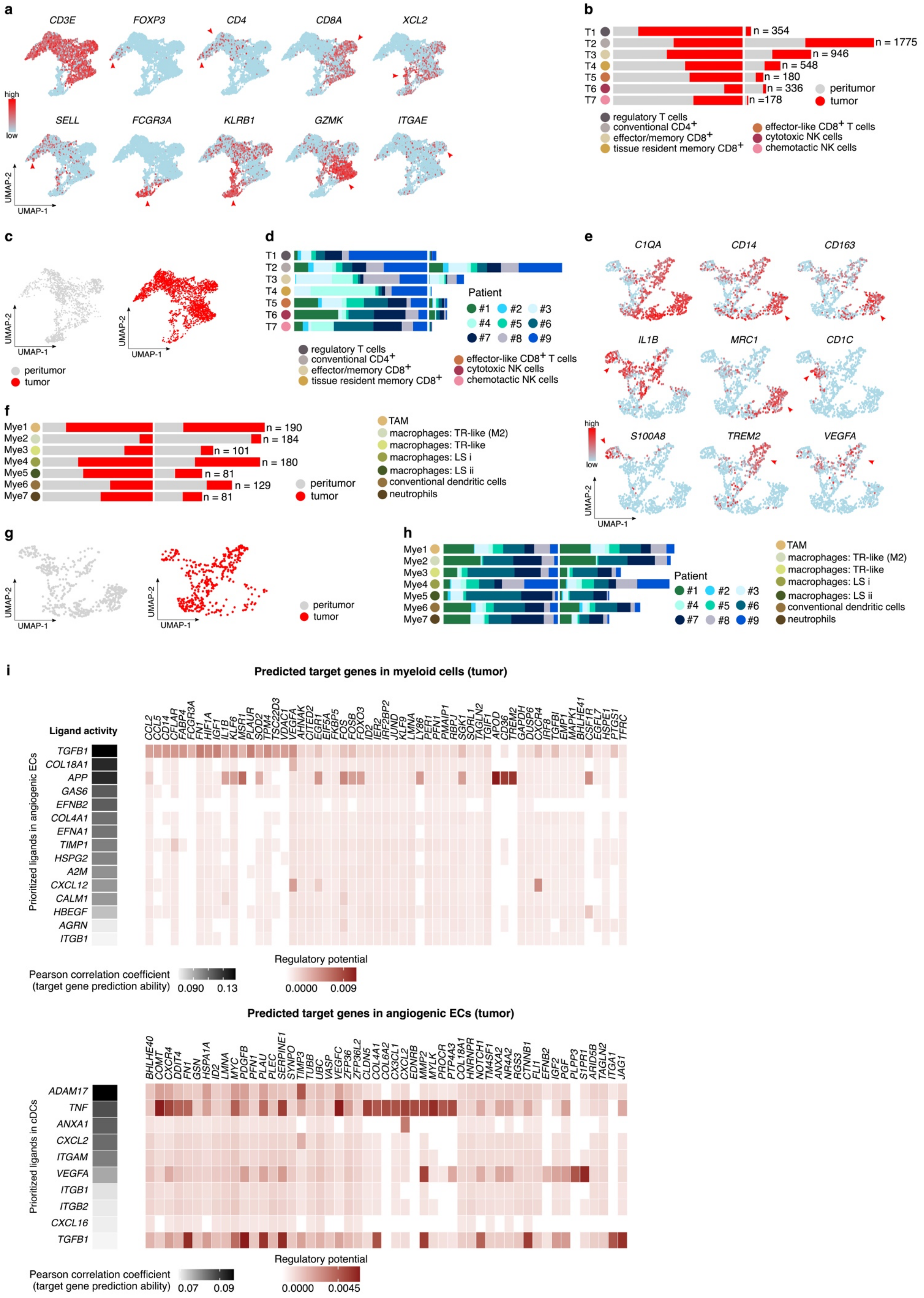

Supplementary Fig. 7: Details on immune cell subclustering and RLI prediction. a UMAP-plots showing expression of representative marker genes in the different T-/NK cell subclusters. Color scale: red – high expression, blue – low expression. Red arrowheads indicate cells highly expressing the marker gene. **b** Contribution of tumoral and peri-tumoral cells to each T-/NK cell subcluster. Left: Relative contribution of each phenotype scaled to 100%. Right panel: contribution of tumoral and peri-tumoral cells in absolute numbers (absolute representation); total number of analyzed cells on the right. NK – natural killer. **c** UMAP-plots of peri-tumoral and tumoral T-/NK cells, color-coded by condition. **d** Contribution of individual patient samples to each T-/NK cell subcluster. Left: relative. Right: absolute. **e** UMAP-plots showing expression of representative marker genes in the different myeloid subclusters. Color scale: red – high expression, blue – low expression. Red arrowheads indicate cells highly expressing the marker gene. **f** Contribution of tumoral and peri-tumoral cells to each myeloid cell subcluster. Left: relative. Right: absolute; total number of analyzed cells on the right. TAM – tumor associated macrophages, TR – tissue resident, LS – lower sequencing depth. **g** UMAP-plots of peri-tumoral and tumoral myeloid cells, color-coded by condition. **h** Contribution of individual patient samples to each myeloid cell subcluster. Left: relative. Right: absolute. **i (Top panel)** NicheNet analysis showing prioritized ligands (left) expressed in angiogenic ECs predicted to regulate target genes in myeloid cells. Ligands were ranked by their likelihood of regulating target genes in tumoral *versus* peri-tumoral samples. Predicted ligand–target matrix denoting the regulatory potential between ligands (y-axis) expressed in angiogenic ECs, and target genes (x-axis) expressed in tumoral myeloid cells is shown on the right. Color scale: dark red/brown, high regulatory potential score; light red/brown, low regulatory potential score. **(Bottom panel)** NicheNet analysis showing prioritized ligands (left) expressed in conventional DCs (cDCs) predicted to regulate target genes in angiogenic ECs. Ligands were ranked by their likelihood of regulating target genes in tumoral *versus* peri-tumoral samples. Predicted ligand–target matrix denoting the regulatory potential between ligands (y-axis) expressed in cDCs, and target genes (x-axis) expressed in tumoral angiogenic ECs is shown on the right. Color scale: dark red/brown, high regulatory potential score; light red/brown, low regulatory potential score.

Supplementary Fig. 8

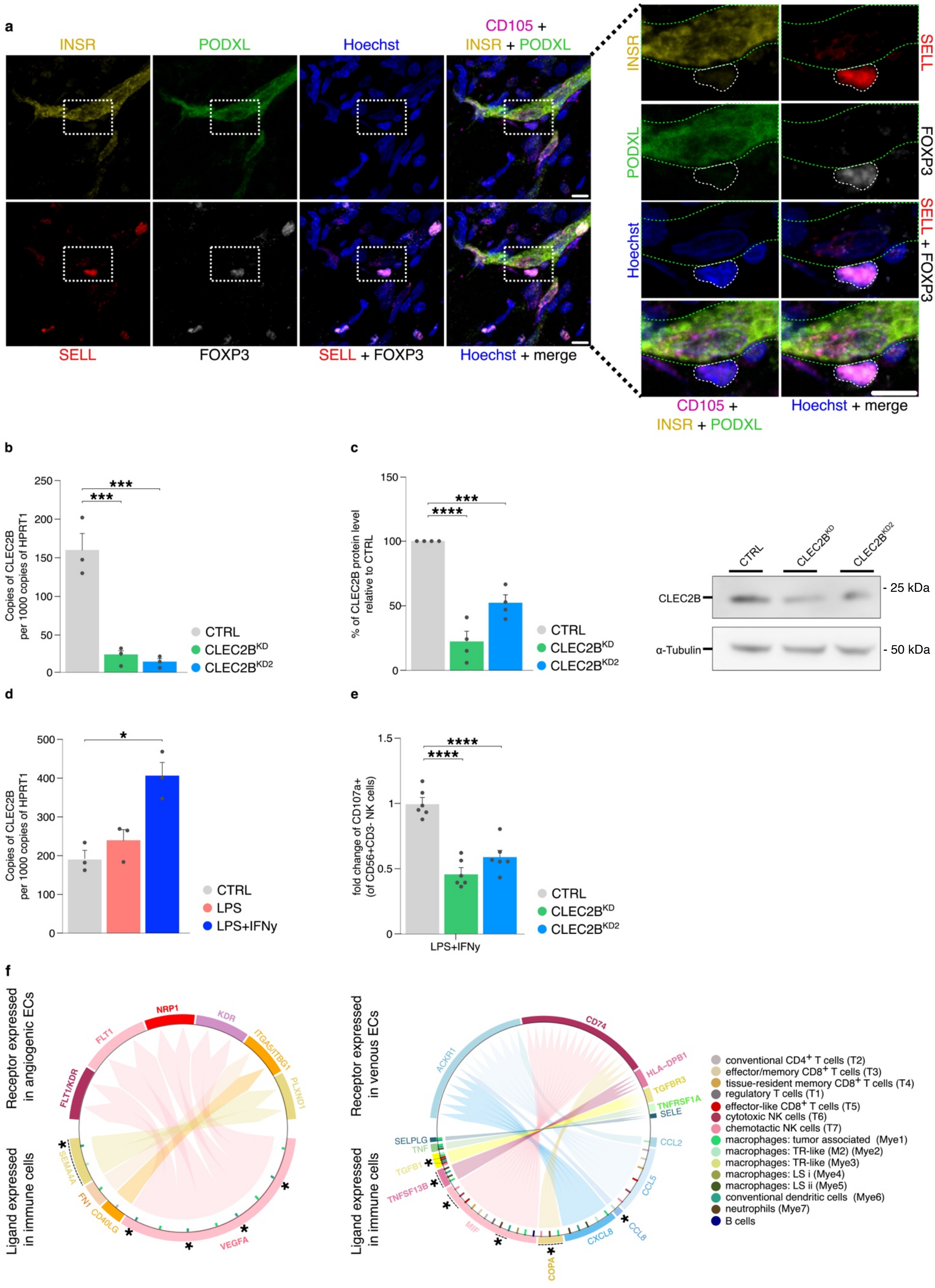

Supplementary Fig. 8: Additional information to the receptor ligand interaction predictions.
a Representative micrographs of human breast tumoral tissue sections, immunostained for CD105 (magenta), FOXP3 (white), INSR (yellow), PODXL (green), SELL (red) and counterstained with Hoechst using Akoya's Opal™ Multiplex IHC system (n=9). Dotted green line delineates ECs, dotted white lines surround FOXP3^{+} cells. Scale bar: 25 µm. Right panels show magnifications of boxed areas. **b** Quantitative real-time PCR showing *CLEC2B* KD efficiency in HUVECs. Color-coded per control or KD-construct (gray PLKO, green CLEC2B^{KD}, blue CLEC2B^{KD2}). Data are mean ± SEM, n=3, ***p<0.001 (exact p-values = 0.0007 and 0.0005, respectively). One-way ANOVA with correction for multiple comparisons (Dunnett's). KD – knock down. **c** Densiometric quantification (left) and representative immunoblot (right) showing CLEC2B KD efficiency at the protein level in HUVECs. Color-coded per control or KD-construct (gray PLKO, green CLEC2B^{KD}, blue CLEC2B^{KD2}). For calculation of the % reduction in CLEC2B expression relative to CTRL, the CLEC2B/housekeeping gene ratio in the CLEC2B^{KD} conditions was determined separately for every HUVEC donor analyzed, and normalized to the CLEC2B/housekeeping gene ratio in the CTRL condition (in the same donor); alpha-Tubulin or GAPDH were used as housekeeping genes. Data are mean ± SEM, n=4, ***p<0.001, ****p<0.0001 (exact p-values= <0.0001 and 0.0003, respectively). One-way ANOVA with correction for multiple comparisons (Dunnett's). **d** Quantitative real-time PCR showing *CLEC2B* expression in HUVECs upon treatment with LPS (pink) and LPS+IFNγ (blue). Data are mean ± SEM, n=3, *p<0.05 (exact p-value=0.0445). One-way ANOVA with correction for multiple comparisons (Dunnett's). **e** Quantification of NK degranulation (as measured by the percentage of CD107a^{+} NK cells) after co-culture for 24h with LPS-IFNγ-treated HUVECs. Color-coded per control or KD-construct (gray PLKO, green CLEC2B^{KD}, blue CLEC2B^{KD2}). Data are mean ± SEM, represented as a fold change, n=6, ****p<0.0001 (exact p-values= <0.0001). One-way ANOVA with correction for multiple comparisons (Dunnett's). **f** Circos plots representing RLI analysis between immune cells and angiogenic/venous ECs. Ligand is expressed on immune cell subclusters, receptor is expressed on angiogenic (left panel) or venous ECs (right panel). Plots are color-coded for receptor-ligand pairs (inner circle, arrows, gene names) and immune cell subclusters expressing the ligand (bars perpendicular to the inner circle). RLI pairs considered novel between ECs and specific immune cell clusters/subtypes are indicated in bold (genes) and with asterisks (subclusters).

Supplementary Fig. 9

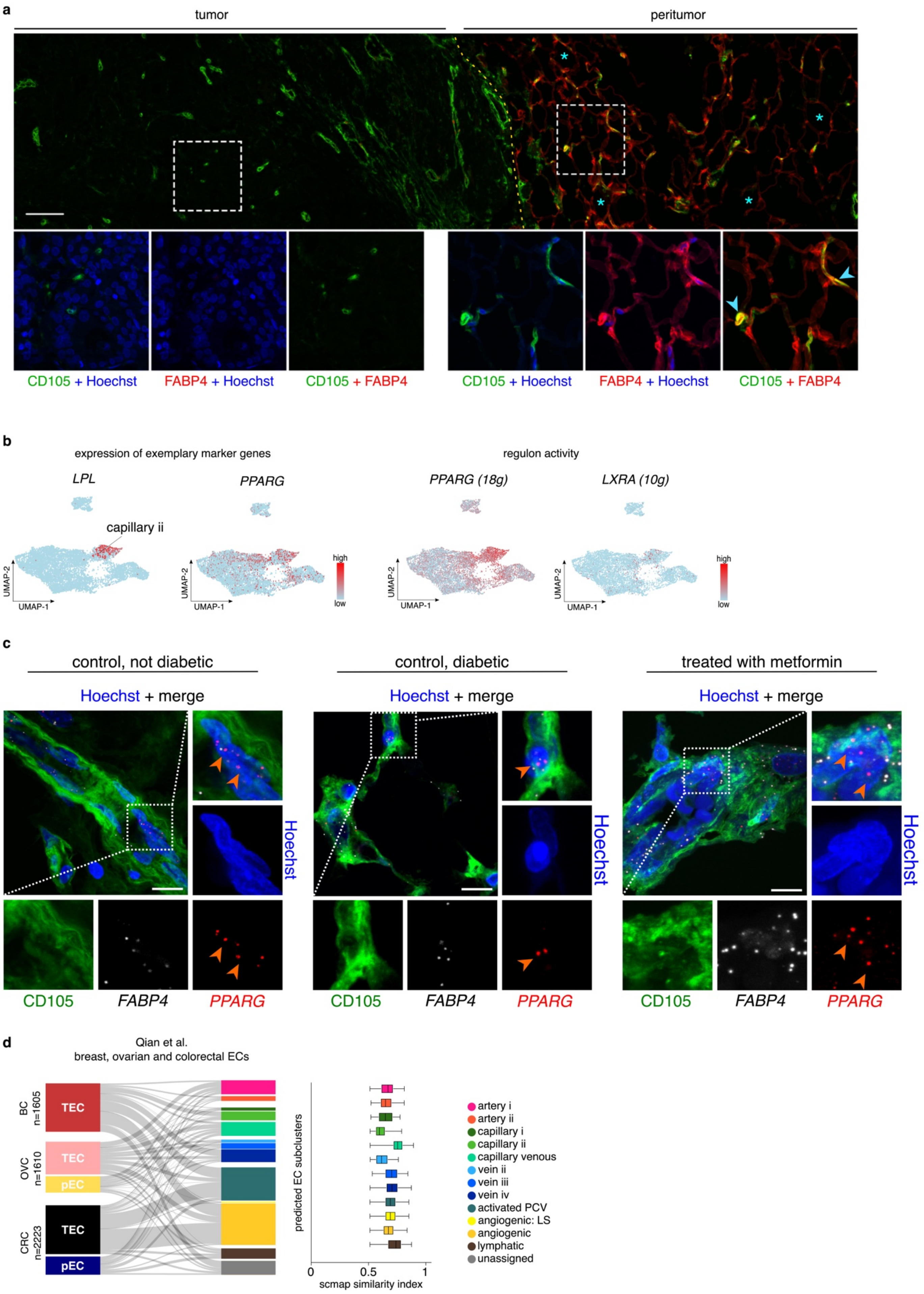

Supplementary Fig. 9: Transcriptomic heterogeneity of breast EC metabolism. a Representative micrographs of human breast tissue on the interphase between tumor (left) and peri-tumor (right), immunostained for CD105, FABP4 and counterstained with Hoechst (n=7). Yellow dotted line indicates putative tumor border. Lower panels: magnifications of the boxed areas in the upper panels. Light blue asterisks indicate representative putative adipocytes, which (besides LIPECs) are also positive for FABP4. Light blue arrows indicate FABP4$^+$ ECs. Scale bar: 100 µm. **b** UMAP-plot of marker gene expression (*LPL, NR1C3 (PPARG)*) (left two panels) or *NR1C3 (PPARG)* and *LXRA* (*NR1H3*) regulon activity predicted by SCENIC analysis (right two panels; numbers between brackets indicate the number of genes within the regulons for the respective transcription factor). Color scale: red – high expression, blue – low expression. **c** Representative micrographs of human breast tumor tissue sections in non-diabetic (left; n=8) or diabetic (middle; n=8) control BC patients and in (diabetic; n=9) BC patients pre-treated with metformin (right), showing combined immunostaining for CD105 with *in situ* hybridization (RNAscope) for *FABP4* and *PPARG* and counterstaining with Hoechst. Right & bottom panels: magnifications of the boxed areas in the main left panels. Orange arrowheads indicate (nuclear) *PPARG* in *FABP4*$^+$ ECs. Scale bar: 10 µm. **d** Sankey diagram (left panel), showing the assignment of EC subclusters (identified in our in-house generated breast EC taxonomy) to unannotated breast cancer (BC), ovarian cancer (OVC) or colorectal cancer (CRC) tumoral (TEC) and normal (peri-tumoral, pEC) ECs from Qian et al. (2020)[23] (n = 5438 ECs). BC, OVC and CRC ECs are visualized on the left; cluster projections are visualized on the right (color-coded according to the breast EC taxonomy subclustering, see legend on the right). Box plots (right panel) depict the scmap similarity index. Boxes extend from the 25th to 75th percentiles, line in the middle of the box is plotted at the median. Whiskers = min and max. LS – lower sequencing depth, PCV – post-capillary venules.

Supplementary Fig. 10

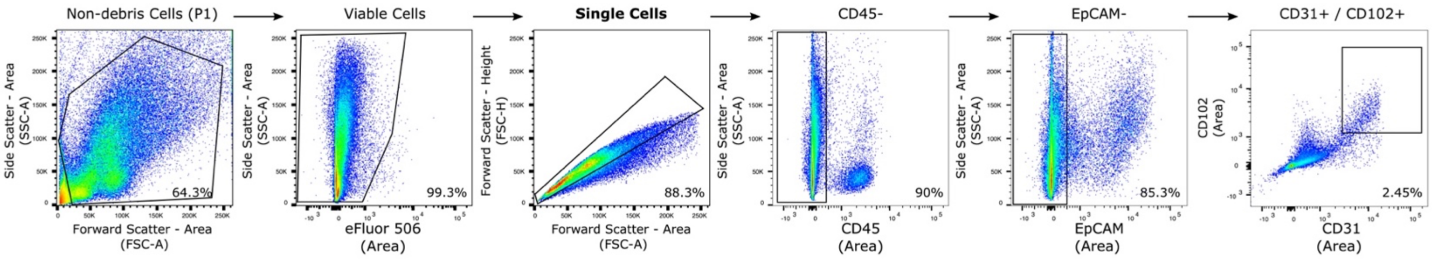

Supplementary Fig. 10: FACS gating strategy for sorting human breast tissue ECs. Representative sequential FACS data and sorting gates for dissociated human breast (cancer) cells. The final sort for the EC-enriched fractions was of viable single cells from the $CD45^-$/$EpCAM^-$/$CD31^+$/$CD102^+$ compartment (right panel). For pME and TME (without EC-enrichment) fractions, viable single cells were sorted (third panel, bold). Percentages reflect the fraction of the (previous) parent population.

Supplementary Table 1

REAGENT or RESOURCE	SOURCE	IDENTIFIER
**Antibodies**		
Anti-α-Smooth Muscle – FITC; dilution 1:100; clone FL-D6	Sigma-Aldrich	Cat#F3777
Anti-α-Tubulin; dilution 1:1000; clone DM1A	Sigma-Aldrich	Cat#T6199
Anti-GAPDH; dilution 1:1000; clone 14C10	Cell Signaling	Cat#2118
Anti-ACKR1; dilution 1:100 (polyclonal)	Sigma-Aldrich	Cat#HPA016421
Anti-AICL (Anti-CLEC2B); dilution 1:25; clone EPR22061	Abcam	Cat#ab221158
Alexa Fluor® 488; dilution 1:1000 Donkey anti-Rabbit IgG (H+L) Highly CrossAdsorbed Secondary Antibody	Thermo Fisher Scientific	Cat# A21206
Alexa Fluor® 647; dilution 1:1000 Donkey anti-Rabbit IgG (H+L) Highly Cross-Adsorbed Secondary Antibody	Thermo Fisher Scientific	Cat# A31573
Anti-CD107a; dilution 1:100; clone H4A3	Biolegend	Cat#328605
Anti-CD16; dilution 1:50; clone 2Q1240	Santa Cruz Biotechnology	Cat#sc-70548
Anti-CD3; dilution 1:100; polyclonal	Agilent Dako	Cat#GA503
Anti-CD3; dilution 1:100; clone HIT3a	Biolegend	Cat#300323
Anti-CD31 Antibody (FITC); dilution 1:100; clone 390	Thermo Fisher Scientific	Cat#11-0311-82
Anti-CD326 (EpCAM) Antibody (PE); dilution 1:500; clone 323/A3	Thermo Fisher Scientific	Cat#MA5-38717
Anti-CD36; dilution 1:100; clone EPR6573	Abcam	Cat#ab133625
Anti-CD45; dilution 1:100; clone 2D1	Biolegend	Cat#368521
Anti-CD45 Antibody (PE-Cy7); dilution 1:700; clone HI30	Thermo Fisher Scientific	Cat#25-0459-42
Anti-CD56; dilution 1:100; clone 5.1H11	Biolegend	Cat#362531
Anti-CD68; dilution 1:100; clone KP1	Agilent Dako	Cat#IR609
Anti-CLEC2B; dilution 1:500; polyclonal	Thermo Fisher Scientific	Cat#PA5-24704
Anti-Cytokeratin 5/6; dilution 1:100; clone D5/16 B4	Agilent Dako	Cat#GA780
Anti-Cytokeratin 18/8; dilution 1:100; clone EP17/EP30	Agilent Dako	Cat#IR094
Anti-Endoglin/CD105; dilution 1:100 for IF; 1:300 for Multiplex Opal system; polyclonal	R&D Systems	Cat#AF1097
Anti-FABP4; dilution 1:500; polyclonal	Abcam	Cat#ab13979
Anti-FOXP3; dilution 1:300; clone 236A/E7	Thermo Fisher Scientific	Cat#14-4777-82
Anti-HLA-DR; dilution 1:100; clone EPR3692	Abcam	Cat#ab92511
Anti-INSR; dilution 1:100 for IF; 1:600 for Multiplex Opal system; polyclonal	Thermo Fisher Scientific	Cat#PA5-27334
Anti-ICAM-2 Antibody (CD102), Alexa Fluor 647; dilution 1:50; clone 3C4	BD Pharmingen™	Cat#564677
Anti-KLRF1; dilution 1:50; polyclonal	Abcam	Cat#ab198928

Anti-L-Selectin/CD62L; dilution 1:100; polyclonal	Abcam	Cat#ab264045
Anti- Podocalyxin; dilution 1:100; polyclonal	R&D Systems	Cat#AF1658
**Biological samples**		
Human breast samples (obtained from UZ Leuven Biobank, post- surgical resection; Medical Ethics Committee UZ/KU Leuven under the protocol S57123)	This paper	N/A
Human umbilical vein endothelial cells (HUVECs)<br>Ethics Committee Research KU Leuven/UZ Leuven (approval number S571230)	This paper	N/A
**Chemicals, Peptides and Recombinant Proteins**		
DNase I	Sigma-Aldrich	Cat#D4527-10KU
ECGS (endothelial cell medium growth supplement mix)	PromoCell	Cat#C-39216
EDTA	Sigma-Aldrich	Cat#ED2P-500G
EGM2 (Endothelial growth medium)	PromoCell	Cat#C-22011
Endothelial cell growth factor supplements (ECGS/Heparin)	PromoCell	Cat#C-30120
Fixable Viability Dye eFluor™ 506	Thermo Fisher Scientific	Cat#65-0866-14
Gelatin from bovine skin	Sigma-Aldrich	Cat#G9391
Hoechst 33258	Sigma-Aldrich	Cat#B2261
IFNγ	Peprotech	Cat#300-02
LPS (lipopolysaccharide)	Sigma	Cat#L2630
Phosphatase inhibitors (PhosSTOP)	Roche	Cat#04906837001
Protease inhibitors (cOmplete, EDTA-free Protease Inhibitor Cocktail)	Roche	Cat#11873580001
RIPA Lysis and Extraction Buffer	Thermo Fisher Scientific	Cat#89901
RPMI	Gibco	Cat#21875034
Sodium pyruvate	Thermo Fisher Scientific	Cat#1360070
SuperSignal West Femto Maximum Sensitivity Substrate	Thermo Fisher Scientific	Cat#34095
TaqMan Fast Universal PCR Master Mix (2X)	Thermo Fisher Scientific	Cat#4364103
Trypsin-EDTA (0.25%), phenol red	Thermo Fisher Scientific	Cat#25200056
**Critical Commercial Assays**		
Akoya's Opal™ Multiplex IHC system	Akoya Biosciences	Cat#NEL821001KT
Chromium i7 Multiplex Kit	10x Genomics	Cat#PN-120262
Chromium Single Cell 3' Library, Gel Bead & Multiplex Kit and Chip Kit, v2	10x Genomics	Cat#PN-120237
Chromium Single Cell A Chip Kit	10x Genomics	Cat#PN-120236
DAB Substrate Kit	Abcam	Cat#ab64238
NK Cell isolation kit, human	Miltenyi Biotec	Cat#130-092-657
Pierce ECL Western Blotting Substrate	Thermo Fisher Scientific	Cat#32106
PureLink RNA Mini Kit	Thermo Fisher Scientific	Cat#12183018A
Multiplex Fluorescent Detection Kit v2	ACDBio	Cat#323110
Script cDNA synthesis kit	Bio-Rad	Cat#1708891
TSA Cyanine 3 (Cy3) System	Perkin Elmer	Cat#NEL704A001KT
TSA Cyanine 5 (Cy5) System	Perkin Elmer	Cat#NEL705A001KT
TSA Fluorescein System	Perkin Elmer	Cat# NEL701A001KT
**Deposited data**		

RNA-sequencing raw and analyzed data human breast EC and ME cells	This paper	GEO: GSE155109
**Oligonucleotides**		
Human CLEC2B qRT-PCR primers	IDT Integrated DNA Technologies	NM_005127 Hs.PT.58.39337866
Human HPRT qRT-PCR primers	IDT Integrated DNA Technologies	NM_000194 Hs.PT.58.2145446
RNAscope Probe Hs ACKR1	ACDBio	Cat#525131-C2
RNAscope Probe Hs FABP4	ACDBio	Cat#470641
RNAscope Probe Hs ID2	ACDBio	Cat#500901
RNAscope Probe Hs PPARG	ACDBio	Cat#441691-C2
RNAscope Probe 3-plex Negative Control Probe	ACDBio	Cat#320871
RNAscope Probe 3-plex Positive Control Probe_Mm	ACDBio	Cat#320881
shRNA CLEC2B (#1), Clone ID: TRCN0000056490	Merck	Cat#NM_005127
shRNA CLEC2B (#2), Clone ID: TRCN0000056492	Merck	Cat#NM_005127
**Other**		
40 µm cell strainer	Sigma-Aldrich	Cat#CLS431750-50EA
100 µm cell strainer	Sigma-Aldrich	Cat#CLS431752-50EA
Antibiotic-antimycotic	Thermo Fisher Scientific	Cat#15240062
Bovine serum albumin (BSA Fraction V)	Sigma-Aldrich	Cat#A3803-50G
Bovine serum albumin (UltraPure BSA)	Thermo Fisher Scientific	Cat#AM2616
Collagenase type I	Thermo Fisher Scientific	Cat#17018029
Collagenase type II	Thermo Fisher Scientific	Cat#17101015
Dispase	Thermo Fisher Scientific	Cat#17105-041
Fetal bovine serum (FBS)	Thermo Fisher Scientific	Cat#16000044
Ficoll"-Paque Plus	GE Life Sciences	Cat#GE17-1440-02
KnockOut^{TM} DMEM	Thermo Fisher Scientific	Cat#10829018
Luna Cell counting Slides	Logos Biosystems	Cat#L12001
MEM NEAA	Thermo Fisher Scientific	Cat#11140035
Nitrocellulose Pre-Cut Blotting Membranes, 0.45 mm pore size	Thermo Fisher Scientific	Cat#LC2001
NuPAGE 10% Bis-Tris Protein Gels, 1.5 mm, 10-well	Thermo Fisher Scientific	Cat#NP0315BOX
NuPAGE 4-12% Bis-Tris Protein Gels, 1.5 mm, 10-well	Thermo Fisher Scientific	Cat#NP0335BOX
NuPAGE LDS Sample Buffer (4X)	Thermo Fisher Scientific	Cat#NP0007
NuPAGE MES SDS Running Buffer (20X)	Thermo Fisher Scientific	Cat#NP0002
NuPAGE MOPS SDS Running Buffer (20X)	Thermo Fisher Scientific	Cat#NP0001
NuPAGE Sample Reducing Agent (10X)	Thermo Fisher Scientific	Cat#NP0009
NuPAGE Transfer Buffer (20X)	Thermo Fisher Scientific	Cat#NP00061
Penicillin/streptomycin	Thermo Fisher Scientific	Cat#15140122
Phosphate buffered saline (DPBS)	Thermo Fisher Scientific	Cat#14190094
ProLong Gold antifade reagent	Thermo Fisher Scientific	Cat#P36934
PVDF Pre-cut Blotting Membranes, 0.2 mm pore size	Thermo Fisher Scientific	Cat#S9549
SOD-peg	Thermo Fisher Scientific	Cat#S9549

Surgical Scalpel Blade No 10	Swann Morton	Cat#0201
**Software and Algorithms**		
BIOMEX	[28]	https://www.vibcancer.be/software-tools/BIOMEX
CellPhoneDB; version 2.0.0	[29]	https://www.cellphonedb.org/explore-sc-rna-seq
Cell Ranger; version 2.2.0	10x Genomics	(tenx; RRID: SCR_01695)
circlize; version 0.4.8	[30]	https://cran.r-project.org/web/packages/circlize/circlize.pdf
clusterProfiler; version 3.6.0	Bioconductor[31]	(clusterProfiler, RRID: SCR_016884)
Fiji (ImageJ)	Open source	https://fiji.sc
GraphPad Prism8, version 8.1.1	Graphpad	(GraphPad Prism; RRID: SCR_002798)
InferCNV	[6,7]	R package (version 1.5.0)
NicheNet (*nichenetr* package)	[32]	https://github.com/saeyslab/nichenetr/blob/master/vignettes/seurat_steps.md.
qvalue: version 2.10.0	[33]	http://github.com/jdstorey/qvalue
pvclust: version 2.0.0	[34]	https://cran.r-project.org/src/contrib/Archive/ pvclust/
scmap; version 1.1.5	[35]	(scmap; RRID: SCR_017338)
Seurat; version 2.3.4	[36]	(Seurat; RRID: SCR_016341)
R version 3.4.4	The R Foundation	https://www.r-project.org
SCENIC; version 1.5.0	[37]	https://aertslab.org/#scenic
SCORPIUS; version 1.0.2	[38]	https://cran.r-project.org/src/contrib/Archive/ SCORPIUS/
RStudio (1.1.456)	Open source	https://rstudio.com
umap: version 0.2.0.0	McInnes et al., 2018	https://cran.r-project.org/src/contrib/Archive/ umap/
VennDiagram; version 1.6.	[39]	20 (VennDiagram; RRID: SCR_002414)

Supplementary Source Data File

Raw blots used in Supplementary Figure 8c
whole blot

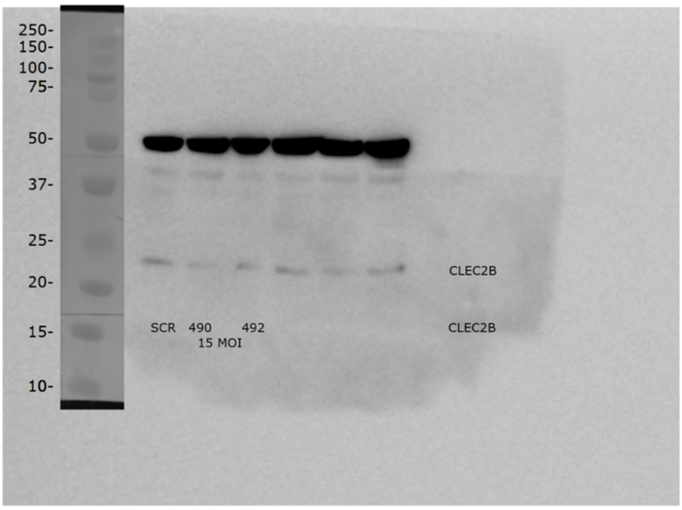

Representative image in Supplementary Figure 8c; cut blot

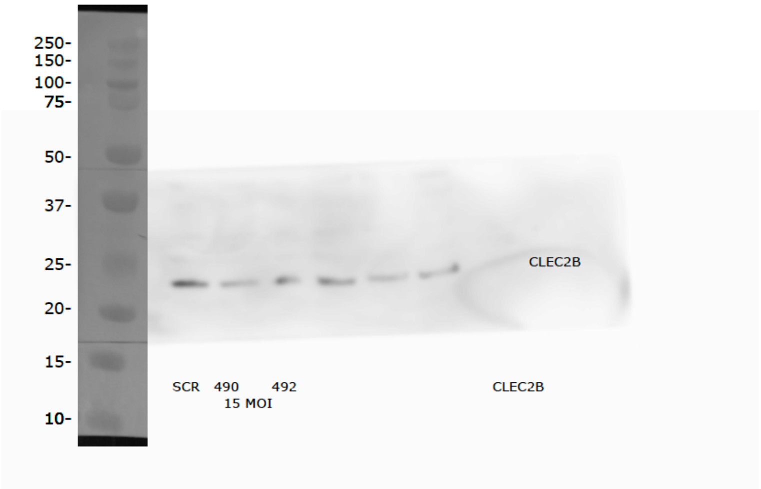

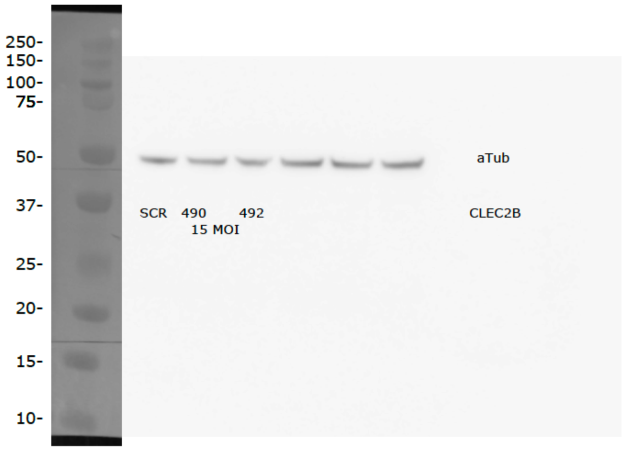

Supplementary References

1 Chou, J., Provot, S. & Werb, Z. GATA3 in development and cancer differentiation: cells GATA have it! *J Cell Physiol* **222**, 42-49 (2010). https://doi.org:10.1002/jcp.21943

2 Iizuka, M. *et al.* Altered intracellular region of MUC1 and disrupted correlation of polarity-related molecules in breast cancer subtypes. *Cancer science* **106**, 307-314 (2015). https://doi.org:10.1111/cas.12596

3 Kumar, B. *et al.* Normal Breast-Derived Epithelial Cells with Luminal and Intrinsic Subtype-Enriched Gene Expression Document Interindividual Differences in Their Differentiation Cascade. *Cancer Res* **78**, 5107-5123 (2018). https://doi.org:10.1158/0008-5472.CAN-18-0509

4 Tasic, B. *et al.* Shared and distinct transcriptomic cell types across neocortical areas. *Nature* **563**, 72-78 (2018). https://doi.org:10.1038/s41586-018-0654-5

5 Wagner, J. *et al.* A Single-Cell Atlas of the Tumor and Immune Ecosystem of Human Breast Cancer. *Cell* **177**, 1330-1345.e1318 (2019). https://doi.org:10.1016/j.cell.2019.03.005

6 Patel, A. P. *et al.* Single-cell RNA-seq highlights intratumoral heterogeneity in primary glioblastoma. *Science (New York, N.Y.)* **344**, 1396-1401 (2014). https://doi.org:10.1126/science.1254257

7 Puram, S. V. *et al.* Single-Cell Transcriptomic Analysis of Primary and Metastatic Tumor Ecosystems in Head and Neck Cancer. *Cell* **171**, 1611-1624.e1624 (2017). https://doi.org:10.1016/j.cell.2017.10.044

8 Berger, A. C. *et al.* A Comprehensive Pan-Cancer Molecular Study of Gynecologic and Breast Cancers. *Cancer cell* **33**, 690-705.e699 (2018). https://doi.org:10.1016/j.ccell.2018.03.014

9 Zhou, S. *et al.* Single-cell RNA-seq dissects the intratumoral heterogeneity of triple-negative breast cancer based on gene regulatory networks. *Molecular Therapy - Nucleic Acids* **23**, 682-690 (2021). https://doi.org:10.1016/j.omtn.2020.12.018

10 Pal, B. *et al.* A single-cell RNA expression atlas of normal, preneoplastic and tumorigenic states in the human breast. *The EMBO Journal* **40**, e107333 (2021). https://doi.org:https://doi.org/10.15252/embj.2020107333

11 Clarke, J. *et al.* Single-cell transcriptomic analysis of tissue-resident memory T cells in human lung cancer. *Journal of Experimental Medicine* **216**, 2128-2149 (2019). https://doi.org:10.1084/jem.20190249

12 Savas, P. *et al.* Single-cell profiling of breast cancer T cells reveals a tissue-resident memory subset associated with improved prognosis. *Nature Medicine* **24**, 986-993 (2018). https://doi.org:10.1038/s41591-018-0078-7

13 Cooper, M. A. *et al.* Human natural killer cells: a unique innate immunoregulatory role for the CD56bright subset. *Blood* **97**, 3146-3151 (2001). https://doi.org:10.1182/blood.V97.10.3146

14 Crinier, A. *et al.* High-Dimensional Single-Cell Analysis Identifies Organ-Specific Signatures and Conserved NK Cell Subsets in Humans and Mice. *Immunity* **49**, 971-986.e975 (2018). https://doi.org:10.1016/j.immuni.2018.09.009

15 Azizi, E. *et al.* Single-Cell Map of Diverse Immune Phenotypes in the Breast Tumor Microenvironment. *Cell* **174**, 1293-1308 e1236 (2018). https://doi.org:10.1016/j.cell.2018.05.060

16 Zilionis, R. *et al.* Single-Cell Transcriptomics of Human and Mouse Lung Cancers Reveals Conserved Myeloid Populations across Individuals and Species. *Immunity* **50**, 1317-1334.e1310 (2019). https://doi.org:10.1016/j.immuni.2019.03.009

17 Cassetta, L. *et al.* Human Tumor-Associated Macrophage and Monocyte Transcriptional Landscapes Reveal Cancer-Specific Reprogramming, Biomarkers, and Therapeutic Targets. *Cancer cell* **35**, 588-602.e510 (2019). https://doi.org:10.1016/j.ccell.2019.02.009

18 Collin, M. & Bigley, V. Human dendritic cell subsets: an update. *Immunology* **154**, 3-20 (2018). https://doi.org:10.1111/imm.12888

19 Chakarov, S. *et al.* Two distinct interstitial macrophage populations coexist across tissues in specific subtissular niches. *Science* **363**, eaau0964 (2019). https://doi.org:10.1126/science.aau0964
20 Mould, K. J., Jackson, N. D., Henson, P. M., Seibold, M. & Janssen, W. J. Single cell RNA sequencing identifies unique inflammatory airspace macrophage subsets. *JCI Insight* **4**, e126556 (2019). https://doi.org:10.1172/jci.insight.126556
21 Chakarov, S. *et al.* Two distinct interstitial macrophage populations coexist across tissues in specific subtissular niches. *Science* **363** (2019). https://doi.org:10.1126/science.aau0964
22 Mould, K. J., Jackson, N. D., Henson, P. M., Seibold, M. & Janssen, W. J. Single cell RNA sequencing identifies unique inflammatory airspace macrophage subsets. *JCI Insight* **4** (2019). https://doi.org:10.1172/jci.insight.126556
23 Qian, J. *et al.* A pan-cancer blueprint of the heterogeneous tumor microenvironment revealed by single-cell profiling. *Cell Res* **30**, 745-762 (2020). https://doi.org:10.1038/s41422-020-0355-0
24 Xie, X. *et al.* Single-cell transcriptome profiling reveals neutrophil heterogeneity in homeostasis and infection. *Nat Immunol* **21**, 1119-1133 (2020). https://doi.org:10.1038/s41590-020-0736-z
25 Travaglini, K. J. *et al.* A molecular cell atlas of the human lung from single-cell RNA sequencing. *Nature* **587**, 619-625 (2020). https://doi.org:10.1038/s41586-020-2922-4
26 Kumar, B. V. *et al.* Human Tissue-Resident Memory T Cells Are Defined by Core Transcriptional and Functional Signatures in Lymphoid and Mucosal Sites. *Cell Rep* **20**, 2921-2934 (2017). https://doi.org:10.1016/j.celrep.2017.08.078
27 Thome, J. J. & Farber, D. L. Emerging concepts in tissue-resident T cells: lessons from humans. *Trends Immunol* **36**, 428-435 (2015). https://doi.org:10.1016/j.it.2015.05.003
28 Taverna, F. *et al.* BIOMEX: an interactive workflow for (single cell) omics data interpretation and visualization. *Nucleic Acids Research* **48**, W385-W394 (2020). https://doi.org:10.1093/nar/gkaa332
29 Efremova, M., Vento-Tormo, M., Teichmann, S.A. & Vento-Tormo, R. CellPhoneDB: inferring cell-cell communication from combined expression of multi-subunit ligand-receptor complexes. Nat Protoc 15, 1484–1506 (2020). https://doi.org/10.1038/s41596-020-0292-x
30 Gu, Z., Gu, L., Eils, R., Schlesner, M. & Brors, B. circlize implements and enhances circular visualization in R. *Bioinformatics* **30**, 2811-2812 (2014). https://doi.org:10.1093/bioinformatics/btu393
31 Yu, G., Wang, L.-G., Han, Y. & He, Q.-Y. clusterProfiler: an R package for comparing biological themes among gene clusters. *OMICS* **16**, 284-287 (2012). https://doi.org:10.1089/omi.2011.0118
32 Browaeys, R., Saelens, W. & Saeys, Y. NicheNet: modeling intercellular communication by linking ligands to target genes. *Nature Methods* **17**, 159-162 (2020). https://doi.org:10.1038/s41592-019-0667-5
33 Storey, C., Cankurtaran, P., Papastathopoulou, P. & Hultink, E. J. Success Factors for Service Innovation: A Meta-Analysis. *Journal of Product Innovation Management* **33**, 527-548 (2016). https://doi.org:https://doi.org/10.1111/jpim.12307
34 Suzuki, R. & Shimodaira, H. Pvclust: an R package for assessing the uncertainty in hierarchical clustering. *Bioinformatics* **22**, 1540-1542 (2006). https://doi.org:10.1093/bioinformatics/btl117
35 Kiselev, V. Y., Yiu, A. & Hemberg, M. scmap: projection of single-cell RNA-seq data across data sets. *Nat Methods* **15**, 359-362 (2018). https://doi.org:10.1038/nmeth.4644
36 Satija, R., Farrell, J. A., Gennert, D., Schier, A. F. & Regev, A. Spatial reconstruction of single-cell gene expression data. *Nat Biotechnol* **33**, 495-502 (2015). https://doi.org:10.1038/nbt.3192
37 Aibar, S. *et al.* SCENIC: single-cell regulatory network inference and clustering. *Nature Methods* **14**, 1083-1086 (2017). https://doi.org:10.1038/nmeth.4463

38 Cannoodt, R. *et al.* SCORPIUS improves trajectory inference and identifies novel modules in dendritic cell development. *bioRxiv*, 079509 (2016). https://doi.org:10.1101/079509

39 Chen, H. & Boutros, P. C. VennDiagram: a package for the generation of highly-customizable Venn and Euler diagrams in R. *BMC bioinformatics* **12**, 35-35 (2011). https://doi.org:10.1186/1471-2105-12-35
