# Supplementary material for: Single cell atlas identifies lipid-processing and immunomodulatory endothelial cells in healthy and malignant breast
Source: Nat Commun. 2022 Sep 20;13:5511. doi: 10.1038/s41467-022-33052-y (PMC9489707; doi:10.1038/s41467-022-33052-y)
Supplement: Supplementary file 2 — Description of Additional Supplementary Files [file 41467_2022_33052_MOESM2_ESM.pdf]

Description of Additional Supplementary Files

File Name: Supplementary Data 1
Description: Clinical characteristics of patients.

File Name: Supplementary Data 2
Description: Quality metrics scRNA & parameters of in silico selection, visualization & clustering.

File Name: Supplementary Data 3
Description: EC clusters: putative function, tabulation of top-ranking marker genes and gene sets.

File Name: Supplementary Data 4
Description: Putative function, tabulation of top-ranking marker genes in major stromal cell clusters.

File Name: Supplementary Data 5
Description: Tabulation of predicted RLI interactions between immune cells and angiogenic/venous ECs.

File Name: Supplementary Data 6
Description: Patient demographics, clinical characteristics, survival, and multivariate Cox regression analysis by study group.
