# Supplementary material for: Single cell atlas identifies lipid-processing and immunomodulatory endothelial cells in healthy and malignant breast
Source: Nat Commun. 2022 Sep 20;13:5511. doi: 10.1038/s41467-022-33052-y (PMC9489707; doi:10.1038/s41467-022-33052-y)
Supplement: Supplementary file 9 — Reporting Summary [file 41467_2022_33052_MOESM9_ESM.pdf]

nature portfolio

Corresponding author(s): Peter Carmeliet

Last updated by author(s): Aug 30, 2022

Reporting Summary

Nature Portfolio wishes to improve the reproducibility of the work that we publish. This form provides structure for consistency and transparency in reporting. For further information on Nature Portfolio policies, see our Editorial Policies and the Editorial Policy Checklist.

Statistics

For all statistical analyses, confirm that the following items are present in the figure legend, table legend, main text, or Methods section.

n/a	Confirmed	
☐	☒	The exact sample size (*n*) for each experimental group/condition, given as a discrete number and unit of measurement
☐	☒	A statement on whether measurements were taken from distinct samples or whether the same sample was measured repeatedly
☐	☒	The statistical test(s) used AND whether they are one- or two-sided<br>*Only common tests should be described solely by name; describe more complex techniques in the Methods section.*
☐	☒	A description of all covariates tested
☐	☒	A description of any assumptions or corrections, such as tests of normality and adjustment for multiple comparisons
☐	☒	A full description of the statistical parameters including central tendency (e.g. means) or other basic estimates (e.g. regression coefficient) AND variation (e.g. standard deviation) or associated estimates of uncertainty (e.g. confidence intervals)
☐	☒	For null hypothesis testing, the test statistic (e.g. *F*, *t*, *r*) with confidence intervals, effect sizes, degrees of freedom and *P* value noted<br>*Give P values as exact values whenever suitable.*
☒	☐	For Bayesian analysis, information on the choice of priors and Markov chain Monte Carlo settings
☒	☐	For hierarchical and complex designs, identification of the appropriate level for tests and full reporting of outcomes
☒	☐	Estimates of effect sizes (e.g. Cohen's *d*, Pearson's *r*), indicating how they were calculated

Our web collection on statistics for biologists contains articles on many of the points above.

Software and code

Policy information about availability of computer code

Data collection	Gene expression matrices were generated using Cell Ranger (10X Genomics, version 2.1.1) using the GRCh38 build of the human reference genome, and further processed using R (version 3.4.4).
Data analysis	Single-cell RNA-sequencing data analysis was performed in R (packages: Seurat v2.3.4, clusterProfiler v3.6.0, qvalue v2.18.0, CellPhoneDB v2.0.0, pvclust v2.2.0, nichenetr v1.0.0, circlize 0.4.8, SCENIC v1.5.0, SCORPIUS v1.0.2, scmap v1.1.5, inferCNV v1.5.0, and BIOMEX version 1.0-4). Immunohistochemical images were processed and analyzed using the Leica MetaMorph AF 1.8 morphometry software package. Densitometric quantifications of Western Blot bands were done with ImageJ. Statistical analysis was performed in either R (version 3.4.4), Graphpad Prism (version 8), or BIOMEX (v 1.0-4).

For manuscripts utilizing custom algorithms or software that are central to the research but not yet described in published literature, software must be made available to editors and reviewers. We strongly encourage code deposition in a community repository (e.g. GitHub). See the Nature Portfolio guidelines for submitting code & software for further information.

Data

Policy information about availability of data

All manuscripts must include a data availability statement. This statement should provide the following information, where applicable:

- Accession codes, unique identifiers, or web links for publicly available datasets
- A description of any restrictions on data availability
- For clinical datasets or third party data, please ensure that the statement adheres to our policy

The raw and processed sequencing data generated in this study have been deposited in the gene expression omnibus database (GEO), under accession code GSE155109 (https://0-www-ncbi-nlm-nih-gov.brum.beds.ac.uk/geo/query/acc.cgi?acc=GSE155109). The publicly available lung (cancer) EC data used in this study are available in the ArrayExpress database at EMBL-EBI under accession code E-MTAB-6308 (https://www.ebi.ac.uk/arrayexpress/experiments/E-MTAB-6308/), and at https://carmelietlab.sites.vib.be/en/software-tools (lung Tumor ECTax). The publicly available breast, ovarian and colorectal (cancer) data used in this study are available in the ArrayExpress database at EMBL-EBI under accession code E-MTAB-8107 (https://www.ebi.ac.uk/arrayexpress/experiments/E-MTAB-8107/), and at https://lambrechtslab.sites.vib.be/en/data-access. The remaining data are available within the Article and Supplementary Information. Source data are provided with this paper.

Human research participants

Policy information about studies involving human research participants and Sex and Gender in Research.

Reporting on sex and gender	All patients analyzed in this study were female.
Population characteristics	For scRNA-seq, all patients were female and hormone receptor-positive/HER2-negative BC; age ranging from 43-78y, cancer stage II or III, histological grade 2 or 3.<br><br>For validation of the clinical cohort: all patients were female and hormone receptor-positive/HER2-negative BC; age ranging from 42-89y, cancer stage I, II or III, histological grade 1,2 or 3, bmi ranging from 17.9-40.4.<br><br>Additional patient characteristics are listed in Supplementary Data 1. TNM staging was done according to the 8th edition of the IUCC guidelines.
Recruitment	Participants were recruited in the multidisciplinary breast clinic by the surgical staff. All patients meeting inclusion criteria (a.o. early breast cancer and primary tumor >/=1cm (thus more likely to yield left-over tissue after surgery and necessary pathology steps)) were considered eligible to participate and were asked for consent.<br>9 hormone sensitive, early stage, treatment-naïve breast cancer patients who underwent resection (broad excision or mastectomy) were recruited by the University Hospital Leuven for single cell RNA-sequencing and protein validation.<br>All patient data used for retrospective clinical cohort analyses (and their validation) were retrieved from the multidisciplinary breast cancer clinic database in the University Hospital Leuven. Participants were not compensated due to the fact that we only used left-over tissue, which did not require additional patient follow-up or contact.
Ethics oversight	All tissue samples were obtained under written informed consent and were fully anonymized. The study was approved by the Medical Ethics Committee UZ Leuven under protocol number S57123. All patient data used for retrospective clinical cohort analyses (and validation) were retrieved from the multidisciplinary breast cancer clinic database in the university hospital Leuven, which was approved by the Medical Ethics Committee UZ Leuven under protocol number S63779.

Note that full information on the approval of the study protocol must also be provided in the manuscript.

Field-specific reporting

Please select the one below that is the best fit for your research. If you are not sure, read the appropriate sections before making your selection.

☒ Life sciences ☐ Behavioural & social sciences ☐ Ecological, evolutionary & environmental sciences

For a reference copy of the document with all sections, see nature.com/documents/nr-reporting-summary-flat.pdf

Life sciences study design

All studies must disclose on these points even when the disclosure is negative.

Sample size	We present single-cell RNA-seq data from breast tissue from 9 early stage, treatment-naïve hormone receptor-positive, HER2-negative breast cancer patients. We furthermore retrospectively analyzed the effect of metformin treatment on the clinical outcome in a cohort of 4,648 female patients with early, hormone receptor-positive/HER2-negative breast cancer who underwent surgery followed by anti-hormonal treatment (thus, tumors with the same setting and molecular characteristics as used in our scRNA-seq cohort). Sample size was determined by the availability of patient samples, and based on previously published studies with a similar scope and focus (e.g. Goveia at al., Cancer Cell 2020, Qian et al., Cell Research 2020).

Data exclusions	For single-cell RNA-seq analysis, the enriched EC samples of patient #6 were not sequenced due to technical reasons, and the enriched pEC sample of patient #1 and pME sample of patient #2 were sequenced but excluded in the analysis after quality control. We used the following quality control steps: genes expressed by <10 cells or with a row mean of <0.003 were not considered, cells expressing <300 genes (low quality), a number of genes >2 standard deviations above the mean (potential doublets), or >10% of unique molecular identifiers (UMIs) derived from the mitochondrial genome were removed. Despite these quality control steps, we identified a few subclusters likely representing doublets in later stages of the analysis/annotation, which were not included in certain downstream analyses (e.g., receptor-ligand interaction predictions). For pseudotime analysis, we only included EC subclusters belonging to different traditional vascular beds that grouped together in the hierarchical clustering analysis as depicted in Fig. 1e: arteries (EC2-3), capillaries (EC11-12), and veins (EC8-10). Vein i (EC6) and vein ii (EC7; patient-specific) were not included in the analysis, as well as the angiogenic ECs (angiogenic (EC4), angiogenic – LS (EC5)), and lymphatic (EC1) ECs. For interactome analysis, EC-self interactions or interactions between EC subclusters and mast cells, plasmacytoid dendritic cells, or plasma cells (all <100 cells per cluster) were excluded. EC subclusters identified in the TME dataset (not EC-enriched) were also not considered for the analysis.<br><br>For the retrospective cohort analysis, 265 patients were not considered for downstream analysis due to lack of critical metadata that could influence the survival analysis.
Replication	All reported findings were replicated across multiple independent biological samples (see figure legends for n).
Randomization	The patients were recruited randomly in this study.
Blinding	Blinding was not necessary/relevant for this study, as we aimed to explore differences between peritumoral and tumoral derived endothelial cells, and we focus on statistical significant differences.

Reporting for specific materials, systems and methods

We require information from authors about some types of materials, experimental systems and methods used in many studies. Here, indicate whether each material, system or method listed is relevant to your study. If you are not sure if a list item applies to your research, read the appropriate section before selecting a response.

Materials & experimental systems

n/a	Involved in the study	
	x	Antibodies
x		Eukaryotic cell lines
x		Palaeontology and archaeology
x		Animals and other organisms
x		Clinical data
x		Dual use research of concern

Methods

n/a	Involved in the study	
x		ChIP-seq
	x	Flow cytometry
x		MRI-based neuroimaging

Antibodies

Antibodies used

Anti-α-Smooth Muscle - FITC Sigma-Aldrich Cat#F3777
Anti-α-Tubulin Sigma-Aldrich Cat# T6199
Anti-ACKR1 Sigma-Aldrich Cat#HPA016421
Anti-AICL (Anti-CLEC2B) Abcam Cat#ab221158
Alexa Fluor® 488 BD Pharmingen™ Cat#558068
Alexa Fluor® 647 Thermo Fisher Scientific Cat#A-31573
Anti-CD107a Biolegend Cat#328605
Anti-CD16 Santa Cruz Biotechnology Cat#sc-70548
Anti-CD3 Aligent Dako Cat#GA503
Anti-CD3 Biolegend Cat#300323
Anti-CD31 Antibody (FITC) Thermo Fisher Scientific Cat#11-0311-82
Anti-CD36 Abcam Cat#ab133625
Anti-CD45 Biolegend Cat#368521
Anti-CD45 Antibody (PE-Cy7) Thermo Fisher Scientific Cat#25-0459-42
Anti-CD56 Biolegend Cat#362531
Anti-CD68 Aligent Dako Cat#IR609
Anti-CLEC2B Fisher Scientific Cat#PA5-24704
Anti-Cytokeratin 5/6 Aligent Dako Cat#GA780
Anti-Cytokeratin 18/8 Aligent Dako Cat#IR094
Anti-Endoglin/CD105 R&D Systems Cat#AF1097
Anti-EpCAM Thermo Fisher Scientific Cat#MA5-38717
Anti-FABP4 Abcam Cat#ab13979
Anti-FOXP3 Thermo Fisher Scientific Cat#14-4777-82
Anti-HLA-DR Abcam Cat#ab92511
Anti-INSR Thermo Fisher Scientific Cat#PA5-27334

Anti-ICAM-2 Antibody (CD102), Alexa Fluor 647 BD Pharmingen™ Cat#564677
Anti-KLRF1 Abcam Cat#ab198928
Anti-L-Selectin/CD62L Abcam Cat#ab264045
Anti- Podocalyxin R&D Systems Cat#AF1658
Donkey anti-Rabbit IgG (H+L) Highly CrossAdsorbed Secondary Antibody, Alexa Fluor™488 (A21206)
Donkey anti-Rabbit IgG (H+L) Highly Cross-Adsorbed Secondary Antibody, Alexa Fluor™ 647 (A31573)

Antibody dilutions as used in the manuscript, as well as clone information can be found in Supplementary Table 1.

Validation

All antibodies are commercially available, with validation procedures/publications described on the following sites of the manufacturers:

Anti-α-Smooth Muscle - FITC Sigma-Aldrich Cat#F3777:
- https://www.sigmaaldrich.com/BE/en/product/sigma/f3777
- Validated for immunohistochemistry (IHC) in paraffin-embedded human tissues by the manufacturer
Anti-α-Tubulin Sigma-Aldrich Cat# T6199:
- https://www.sigmaaldrich.com/BE/en/product/sigma/t6199
- Validated for immuno- or Western Blot using human cells by the manufacturer and in published articles (referenced on company website)
Anti-ACKR1 Sigma-Aldrich Cat#HPA016421:
- https://www.sigmaaldrich.com/BE/en/product/sigma/hpa017672?gclid=Cj0KCQjwxIOXBhCrARIsAL1QFCbwKgY4dKxirRxVCuVIhFAryo2wIVU1AMzkmvnKEQ_NabuZ_a5V7NgaAt56EALw_wcB
- Validated for immunohistochemistry (IHC) in paraffin-embedded human tissues by the manufacturer
Anti-AICL (Anti-CLEC2B) Abcam Cat#ab221158:
- https://www.abcam.com/aicl-antibody-epr22061-ab221158.html#lb
- Validated for immunohistochemistry (IHC) in paraffin-embedded human tissues by the manufacturer
Anti-CD107a Biolegend Cat#328605:
- https://www.biolegend.com/fr-fr/products/fitc-anti-human-cd107a-lamp-1-antibody-4966
- Each lot of this antibody is quality control tested by immunofluorescent staining with flow cytometric analysis by the manufacturer.
Anti-CD16 Santa Cruz Biotechnology Cat#sc-70548:
- validated for IHC in human tissues in https://www.ncbi.nlm.nih.gov/pmc/articles/PMC3488509/
Anti-CD3 Agilent Dako Cat#GA503:
- validated for IHC in paraffin-embedded human tissues in multiple publications, as stated on company web page: https://www.citeab.com/antibodies/3383123-ga503-cd3
Anti-CD3 Biolegend Cat#300323
- https://www.biolegend.com/fr-lu/products/alexa-fluor-700-anti-human-cd3-antibody-3417
- Each lot of this antibody is quality control tested by the manufacturer by immunofluorescent staining with flow cytometric analysis.
Anti-CD31 Antibody (FITC) Thermo Fisher Scientific Cat#11-0311-82
- Tested tested by immunofluorescent staining with flow cytometric analysis, as stated on company web page: https://www.thermofisher.com/antibody/product/CD31-PECAM-1-Antibody-clone-390-Monoclonal/11-0311-82
Anti-CD36 Abcam Cat#ab133625
- https://www.abcam.com/cd36-antibody-epr6573-ab133625.html#lb
- Validated for IHC in paraffin-embedded human tissues by the manufacturer
Anti-CD45 Biolegend Cat#368521
- https://www.biolegend.com/de-at/products/brilliant-violet-421-anti-human-cd45-antibody-14686
- Each lot of this antibody is quality control tested by the manufacturer by immunofluorescent staining with flow cytometric analysis.
Anti-CD45 Antibody (PE-Cy7) Thermo Fisher Scientific Cat#25-0459-42
- https://www.thermofisher.com/antibody/product/CD45-Antibody-clone-HI30-Monoclonal/25-0459-42
- Validated for immunostaining and flow cytometric analysis on human cells by the manufacturer
Anti-CD56 Biolegend Cat#362531:
- https://www.biolegend.com/nl-nl/products/brilliant-violet-650-anti-human-cd56-ncam-antibody-11753
- Each lot of this antibody is quality control tested by the manufacturer by immunofluorescent staining with flow cytometric analysis.
Anti-CD68 Agilent Dako Cat#IR609:
- Validated for IHC in paraffin-embedded human tissues: https://www.citeab.com/antibodies/3383149-ir609-cd68-autostainer-link-48
Anti-CLEC2B Fisher Scientific Cat#PA5-24704
- Validated for Western Blot in human cells: https://www.thermofisher.com/antibody/product/CLEC2B-Antibody-Polyclonal/PA5-24704
Anti-Cytokeratin 5/6 Agilent Dako Cat#GA780:
- https://www.agilent.com/en/product/immunohistochemistry/antibodies-controls/primary-antibodies/cytokeratin-5-6-%28dako-omnis%29-76275
- The Dako FLEX Ready-to-Use (RTU) primary antibodies work on formalin-fixed, paraffin-embedded tissue sections as a set of dedicated reagents for clinical routine diagnostics.
Anti-Cytokeratin 18/8 Agilent Dako Cat#IR094
- https://www.agilent.com/store/en_US/Prod-IR09461-2/IR09461-2
- The Dako FLEX Ready-to-Use (RTU) primary antibodies work on formalin-fixed, paraffin-embedded tissue sections as a set of dedicated reagents for clinical routine diagnostics.
Anti-EpCAM Thermo Fisher Scientific Cat#MA5-38717
- https://www.thermofisher.com/antibody/product/EpCAM-CD326-Antibody-clone-323-A3-Monoclonal/MA5-38717
- Validated for immunostaining and flow cytometric analysis on human cells by the manufacturer

Anti-Endoglin/CD105 R&D Systems Cat#AF1097
- https://www.rndsystems.com/products/human-endoglin-cd105-antibody_af1097
- Knockout validated by manufacturer
Anti-FABP4 Abcam Cat#ab13979
- https://www.abcam.com/fabp4-antibody-ab13979.html
- Manufacturer covers the use of ab13979 in IHC on formalin-fixed, paraffin-embedded tissue sections
Anti-FOXP3 Thermo Fisher Scientific Cat#14-4777-82
- https://www.thermofisher.com/antibody/product/FOXP3-Antibody-clone-236A-E7-Monoclonal/14-4777-82
- Validated for IHC in paraffin-embedded human tissues
Anti-HLA-DR Abcam Cat#ab92511
- Validated for IHC in human tissue sections in: https://www.ncbi.nlm.nih.gov/pmc/articles/PMC7868549/
Anti-INSR Thermo Fisher Scientific Cat#PA5-27334
- https://www.thermofisher.com/antibody/product/INSR-Antibody-Polyclonal/PA5-27334
- Validated for IHC in paraffin-embedded human tissues
Anti-ICAM-2 Antibody (CD102), Alexa Fluor 647 BD Pharmingen™ Cat#564677
- https://www.bdbiosciences.com/zh-cn/products/reagents/flow-cytometry-reagents/research-reagents/single-color-antibodies-ruo/apc-mouse-anti-human-cd102.564677
- Validated for immunofluorescent staining with flow cytometric analysis on human cells by manufacturer
Anti-KLRF1 Abcam Cat#ab198928
- https://www.abcam.com/klrf1-antibody-ab198928.html
- Manufacturer covers the use of ab198928 in IHC on formalin-fixed, paraffin-embedded tissue sections
Anti-L-Selectin/CD62L Abcam Cat#ab264045
- https://www.abcam.com/cd62l-antibody-ab264045.html
- Manufacturer covers the use of ab264045 in IHC on formalin-fixed, paraffin-embedded tissue sections
Anti- Podocalyxin R&D Systems Cat#AF1658
- https://www.rndsystems.com/products/human-podocalyxin-antibody_af1658
- Knockout validated and tested for IHC on formalin-fixed, paraffin-embedded tissue sections by manufacturer

Flow Cytometry

Plots

Confirm that:

- [x] The axis labels state the marker and fluorochrome used (e.g. CD4-FITC).
- [x] The axis scales are clearly visible. Include numbers along axes only for bottom left plot of group (a 'group' is an analysis of identical markers).
- [x] All plots are contour plots with outliers or pseudocolor plots.
- [x] A numerical value for number of cells or percentage (with statistics) is provided.

Methodology

Sample preparation

Following surgical resection, samples from the invasive tumor front and adjacent healthy breast tissue (as far away from the tumor border as possible) were taken. Samples were minced into <1mm3 pieces and enzymatically digested in a 7ml digestion medium (KnockOut DMEM, supplemented with penicillin/streptomycin (1x), Antibiotic-Antimycotic (2x), sodium pyruvate (1mM), MEM NEAAs (1x) (Thermo Fisher Scientific), heparin (10U/ml)/ECGF (PromoCell), 0.3%collagenase I, 0.1% collagenase II (Thermo Fisher Scientific), DNase (7.5µM) (Sigma-Aldrich) and dispase (0.25U/ml) (Thermo Fisher Scientific)). Samples were incubated at 37°C for approximately 60 minutes. To improve digestion, samples were pipetted up and down every 10 minutes. Next, 7.5ml of cold PBS containing 0.5% bovine serum albumin (BSA, Sigma-Aldrich) and 2µM EDTA were added and the samples were filtered using a 100µm strainer (Corning). Following centrifugation (300g) at room temperature (RT) for 4 minutes, the supernatant was discarded and cells were resuspended in cold PBS containing 0.5% BSA (Sigma-Aldrich) and 2µM EDTA for further downstream applications.

Instrument

FACS Aria III (BD Biosciences)

Software

FlowJo software (BD Biosciences), version 10.8.1

Cell population abundance

On average, ECs comprised 3-6% of viable, CD45-negative, EpCAM-negative breast (cancer) cells. For EC-enriched fractions, very small populations of plasma (JCHAIN-positive) and red blood cell (HBB-positive) were detected in the resulting scRNA-sequencing data, together comprising 8.9% of the total EC-enriched population.

Gating strategy

Single-cell suspensions were stained with viability dye (VD, eFluor™ 506, dilution 1:1000) and fluorescent antibodies (CD45 (PE-Cy7, dilution 1:700), EpCAM (PE, dilution 1:500), CD31 (AF488, dilution 1:100) and CD102/ICAM2 (APC, dilution 1:50)) for 30 minutes. Then, we FACS-sorted viable single cells (VD–) into low-bind Eppendorf tubes containing 200 µL pure FBS and divided each sample into 2 fractions: one was used to enrich ECs (CD45-, EpCAM-, CD31+, CD102+ cells) and the other one contained all stromal cells. We based the selection of ECs both on CD31 and CD102 to increase purity. See supplementary Figure 10 for a representative overview of the gating strategy.

- [x] Tick this box to confirm that a figure exemplifying the gating strategy is provided in the Supplementary Information.
